# *Pseudomonas* Species Diversity Along the Danube River Assessed by *rpoD* Gene Sequence and MALDI-TOF MS Analyses of Cultivated Strains

**DOI:** 10.3389/fmicb.2020.02114

**Published:** 2020-09-02

**Authors:** Magdalena Mulet, María Montaner, Daniela Román, Margarita Gomila, Clemens Kittinger, Gernot Zarfel, Jorge Lalucat, Elena García-Valdés

**Affiliations:** ^1^Microbiologia, Departament de Biologia, Edifici Guillem Colom, Universitat de les Illes Balears, Palma de Mallorca, Spain; ^2^Diagnostic and Research Institute of Hygiene, Microbiology and Environmental Medicine, Medical University of Graz, Graz, Austria; ^3^Institut Mediterrani d’Estudis Avançats (IMEDEA, CSIC-UIB), Palma de Mallorca, Spain

**Keywords:** *Pseudomonas*, Danube, identification, MALDI-TOF MS, *rpoD*, phylogeny

## Abstract

A collection of 611 *Pseudomonas* isolated from 14 sampling sites along the Danube River were identified previously by MALDI-TOF MS with the VITEK MS system and were grouped in 53 clusters by their main protein profiles. The strains were identified in the present study at the phylospecies level by *rpoD* gene sequencing. Partial sequences of the *rpoD* gene of 190 isolates representatives of all clusters were analyzed. Strains in the same MALDI-TOF cluster were grouped in the same phylospecies when they shared a minimum 95% similarity in their *rpoD* sequences. The sequenced strains were assigned to 34 known species (108 strains) and to 32 possible new species (82 strains). The 611 strains were identified at the phylospecies level combining both methods. Most strains were assigned to phylospecies in the *Pseudomonas putida* phylogenetic group of species. Special attention was given to 14 multidrug resistant strains that could not be assigned to any known *Pseudomonas* species and were considered environmental reservoir of antibiotic resistance genes. Coverage indices and rarefaction curves demonstrated that at least 50% of the *Pseudomonas* species in the Danube River able to grow in the isolation conditions have been identified at the species level. Main objectives were the confirmation of the correlation between the protein profile clusters detected by MALDI-TOF MS and the phylogeny of *Pseudomonas* strains based on the *rpoD* gene sequence, the assessment of the higher species discriminative power of the *rpoD* gene sequence, as well as the estimation of the high diversity of *Pseudomonas* ssp. along the Danube river. This study highlights the *Pseudomonas* species diversity in freshwater ecosystems and the usefulness of the combination of MALDI-TOF mass spectrometry for the dereplication of large sets of strains and the *rpoD* gene sequences for rapid and accurate identifications at the species level.

## Introduction

The Danube River is the second longest river in Europe, 2,800 km crossing nine countries (Germany, Austria, Slovakia, Hungary, Croatia, Serbia, Bulgaria, Ukraine, and Romania). In 2013 the International Commission for the Protection of the Danube River organized the Joint Danube Survey 3 (JDS3). Part of the JDS3 was the assessment of the role of the Danube River as dissemination system of antibiotic resistant microorganisms (Kittinger et al., 2016a). In this context 611 *Pseudomonas* strains were isolated from Danube River samples on Endo Agar, Xylose Lysine Deoxycholat Agar (XLDagar), and Chromocult Coliform Agar (CCA) (all Merck, Austria) and were analyzed according to their antibiotics resistances (Kittinger et al., 2016b). Growth conditions were 37 ± 1°C for 18–24 h selecting for strains able to grow at this temperature. The transfer of antimicrobial resistance genes between environmental *Pseudomonas* has been studied previously, not only in the most representative pathogen, *P. aeruginosa*, but also in other low pathogenic species, like *P. putida* (Peter et al., 2017) or other opportunistic pathogens like *P. stutzeri* (Carvalho-Assef et al., 2010). Susceptibility against ten antibiotics was tested for all isolates and results indicated the presence of human induced resistance and multi-drug resistant (MDR) strains. The 611 *Pseudomonas* isolates were described and identified at the species level by MALDI-TOF mass spectrometry (MALDI-TOF MS) using a commercial database (VITEK MS system; BioMérieux, France) designed mainly for clinical isolates: 405 (66.3%) isolates were identified as *Pseudomonas putida* and 162 (26.5%) as *Pseudomonas fluorescens* (Kittinger et al., 2016b). Nine other *Pseudomonas* species detected were represented by less than 12 isolates each.

The correct species identification of environmental and clinical strains is essential for estimating the intrinsic antibiotic resistance patterns and to study the possible transfer of resistance genes in the environment. The taxonomy of species in the genus *Pseudomonas* is complex, comprising more than 220 different species (Lalucat et al., 2020), and many species, potentially pathogenic, may be found in diverse aquatic environments, from temperate rivers (Pirnay et al., 2005; Sánchez et al., 2014; Kittinger et al., 2016b) to aquatic tropical sediments (Devarajan et al., 2017) or drinking waters (Vaz-Moreira et al., 2012; Sala-Comorera et al., 2016a, b). Therefore, the identification at the species level can be difficult. MALDI-TOF MS is a convenient and rapid method, but the database has to include all species type strains and it is not always discriminative enough at the species level (Mulet et al., 2012). The *rpoD* gene sequence was selected for identifications at the species level, because it has been proven in previous publications that it is a good and reliable tool for species differentiation in the genus *Pseudomonas* (Mulet et al., 2009; Peter et al., 2017). It has been applied previously for the identification of strains isolated from many different environments or has been applied even for culture-independent approaches (Mulet et al., 2011; Sánchez et al., 2014).

In previous publications, we have defined phylospecies (PS) and genomospecies (GS) within the genus *Pseudomonas*. A phylospecies is constituted by strains that share at least 95–96% identity in their partial *rpoD* nucleotide sequences; strains in the same genomospecies share at least 95–96% in their average nucleotide identity values based on BLAST (ANIb) and at least 70% in their genome to genome distance comparisons (GGDC) (Mulet et al., 2012; Gomila et al., 2015). There is a very good correlation between phylospecies and genomospecies (Gomila et al., 2015); all strains in the same genomospecies belong to the same phylospecies based on the *rpoD* partial sequence.

The main purposes are: (1) the demonstration of the superiority of the *rpoD* gene sequence over MALDI TOF MS for the accurate identification of *Pseudomonas* species; (2) the utility of the combination of MALDI-TOF MS to dereplicate large sets of isolates and later sequencing the *rpoD* gene of representatives of the MALDI-TOF MS groupings for identifications as a rapid and precise procedure; (3) the assessment of the *Pseudomonas* species diversity along the Danube river by the identification of a large set of *Pseudomonas* isolates, classifying them in known and potential new species; and (4) to demonstrate the transfer of antibiotics resistances to environmental, autochthonous strains of known species and others considered potential new species. Four hundred strains were assigned to known or new phylospecies and 200 were assigned to phylogenetic groups or subgroups within the genus.

## Materials and Methods

### Sampling Locations

The International Commission for the Protection of the Danube River organized the JDS (Peter et al., 2017) campaign (Kittinger et al., 2016b). Sixty-eight water samples were collected from August 12 to September 26 of 2013. The sampling locations studied in this study were 14 sites along the river: JDS02, JDS03, JDS08, JDS10, JDS22, JDS28, JDS36, JDS38, JDS49, JDS57, JDS59, JDS63, JDS067, JDS68, and are indicated in Supplementary Table S1. Water samples (0.5 ml) were plated on Endo Agar, Lysine Deoxycholate Agar and Chromocult Coliform Agar and were incubated at 37^*o*^C for 18–24 h as described (Kittinger et al., 2016b).

### Bacterial Strains and Growth Conditions

All *Pseudomonas* strains (611) isolated in the Joint Danube Survey 3 (JDS3) campaign identified by MALDI-TOF MS (Kittinger et al., 2016b) and stored at the collection of the Institute of Hygiene, Microbiology and Environmental Medicine, Medical University of Graz isolated at the JDS3 campaign were sent to the Microbiology laboratory of the University of the Balearic Islands (UIB). They were checked for viability and purity after culturing on LB medium (Conda) at 30°C for 24–48 h. To standardize procedures at the UIB laboratory the strains were cultured routinely at 30°C.

### Selection of Strains

An UPGMA dendrogram using the MALDI-TOF MS protein profiles of the 611 strains was constructed with the raw Biomérieux data obtained with the VITEK MS identification system in order to group them in putative species. To obtain the spectra, a colony from blood agar plate (incubated at 37°C) was directly spotted on the MALDI plate, and then overlaid with 1 μl of matrix solution and air-dried. The loaded plate was then placed in the instrument according to the manufacturer’s instructions (Kittinger et al., 2016a). The list of peaks obtained from each mass spectrum was converted to a matrix of 0 and 1, according to the absence or presence of a specific peak. Peaks that differed in less than 1 unit of m/Z between them were considered the same protein. From a list of 6,870 peaks, only 747 different major proteins were considered. The matrix was used to calculate the Pearson distance between all pairs of data and to construct an UPGMA dendrogram. Hundred and ninety strains were selected as representatives of the 53 clearly defined clusters of strains that were at least 69% similar in the dendrogram.

### DNA Extraction, *rpoD* and 16S rRNA Genes PCR Amplification, DNA Sequencing Conditions

Strains were cultured overnight in 4 ml LB (Conda) at 30°C. The DNA extraction, PCR amplification, primers used, purification of the amplified products and DNA sequencing conditions, as well as the sequence analysis procedures, have been previously described (Mulet et al., 2012). PCR conditions for the 16S rRNA gene are described in Mulet et al. (2008). Identifications were performed at the EzTaxon database.

### Phylogenetic Analysis

The partial sequences of the *rpoD* gene (708 nucleotides) of 190 strains representatives of the 53 clusters were aligned and compared with the 220 *Pseudomonas* species type strains *rpoD* nucleotide sequences of an indoor database at the laboratory of Microbiology of the University of the Balearic Islands. The database was updated with all *Pseudomonas* species type strains described until January 2020. The neighbor joining (NJ) (Saitou and Nei, 1987), maximum likelihood (ML) (Felsenstein, 1981) and maximum parsimony (MP) algorithms were used for the comparisons with the integrated tool MEGA5 (Nei and Kumar, 2000; Tamura et al., 2011). A neighbor joining tree (Saitou and Nei, 1987) based on the *rpoD* nucleotide sequences of the 190 representative strains isolated from the Danube River was constructed. Detailed analysis of the specific *Pseudomonas* groups of species, like *P. putida* or *P. stutzeri*, included also additional strains to identify more precisely the strains or to propose them as putative new species. Each different sequence was considered a single phylotype, and several phylotypes were grouped in a single phylospecies when they affiliated in the same phylogenetic branch and the similarity in the *rpoD* gene sequence was equal or greater than 95% (Mulet et al., 2009; Scotta et al., 2013). Phylospecies was abbreviated PS.

### Statistical Analysis

Sequence information obtained was used to calculate phylospecies coverage with the formula $C=1-\left(\frac{n}{N}\right)$ where *C* is the coverage index, *n* the number of singletons (phylospecies with only 1 representative) and *N* the total number of strains. Rarefaction curves were constructed with the PAST software package version 3.20 (Hammer et al., 2001).

## Results

### MALDI-TOF MS Groupings

**FIGURE 1:**
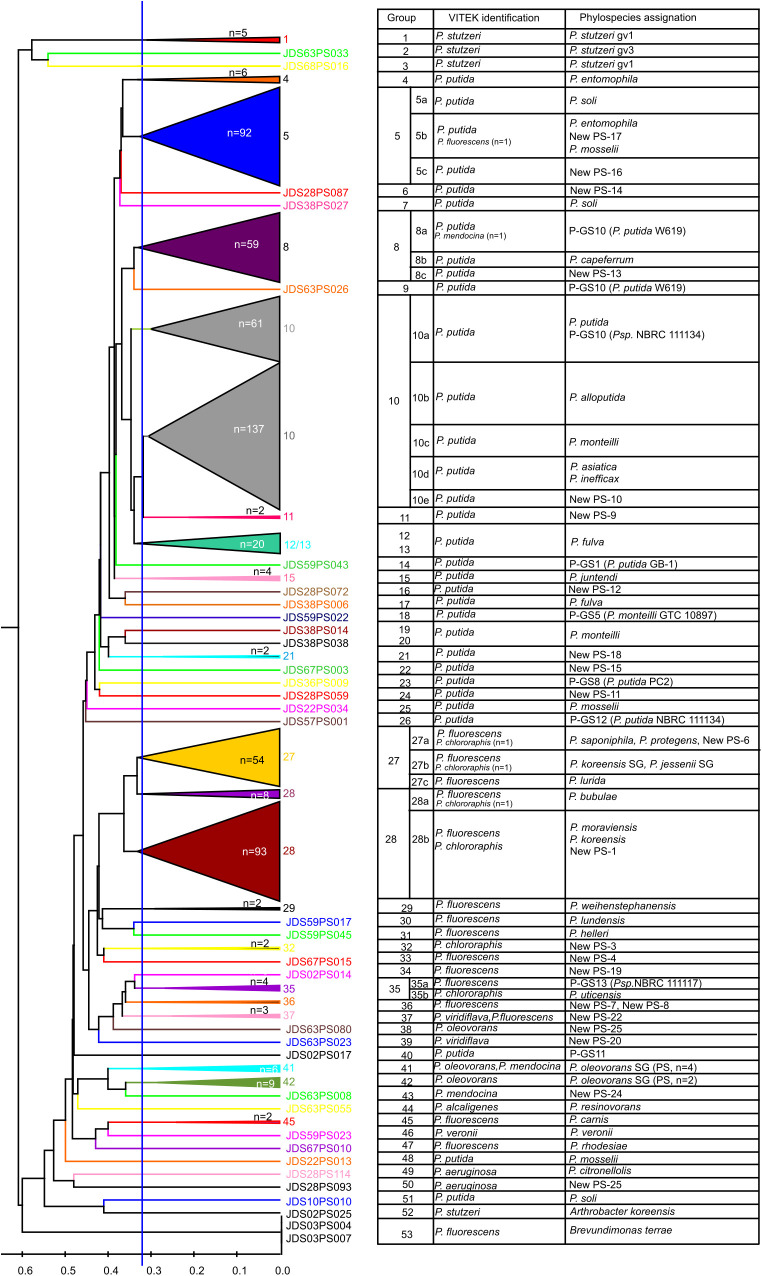
UPGMA dendrogram of the 611 isolates based on the similarities of the MALDI-TOF MS protein profiles, their VITEK identification and assignation to phylospecies by *rpoD* sequencing. On the basis of the *rpoD* sequences, several groups are subdivided and labeled with letters (a, b, and c, etc.).

The 611 isolates were classified in 53 similarity clusters by their MALDI-TOF MS protein profiles as depicted in Figure 1, Table 1, and Supplementary Figure S1. Isolates in each cluster were at least 69% similar in their protein profiles and each cluster was numbered, from 1 to 53. Six groupings comprised a high number of isolates (from 54 to 137), while the others included only 1 to 20 isolates. Isolates identifications with the VITEK MS system were coincident in most cases with the main clusters: groups 1–3 (7 strains) to *P. stutzeri*; groups 4–26 (401 isolates) to *P. putida*; groups 27–33 (161 isolates) to *P. fluorescens*; groups 34–39 (13 isolates) to *P*. *fluorescens* or *P. viridiflava*; groups 41–43 (16 isolates) to *P. mendocina* or *P. oleovorans*; less abundant groups 44–53 (12 isolates) included isolates identified in 5 different species. Some of the more abundant groupings, based on the *rpoD* gene sequence analyses could be identified in more than one phylospecies and were subdivided later in subgroups labeled a, b, c, d, and e.

**TABLE 1:** Groupings of strains based on the MALDI-TOF MS protein profiles and on the *rpoD* sequence analyses and their assignation to phylospecies.

Phylospecies	WC-MALDI-TOF MS (VITEK) identification	WC-MALDI-TOF MS group/Subgroup^*a*^	Number of isotates analyzed by *rpoD*	Number of isolates assigned to PS
*P. fluorescens* SG				
*P. carnis*	*P. fluorescens*	45	1	2
*P. lurida*	*P. fluorescens*	27c	2	3
*P. veronii*	*P. veronii*	46	1	1
*P. rhodesiae*	*P. fluorescens*	47	1	1
*P. koreensis* SG				
*P. koreensis*	*P. fluorescens*	28b	4	4
*P. moraviensis*	*P. fluorescens*	28b	14	14
New PS-1	*P. fluorescens*	28b	1	1
*P. jessenii* SG				
*P. mohnii*	*P. fluorescens*	27b	1	1
*P. corrugata* SG				
*P. kilonensis*	*P. fluorescens*	27b	1	1
*P. rhizophila*	*P. fluorescens*	27b	1	1
New PS-2	*P. fluorescens*	27b	1	1
New PS-3	*P. chlororaphis*	32	2	2
New PS-4	*P. fluorescens*	33	1	1
New PS-5	*P. chlororaphis/fluorescens*	27b	1	1
*P. protegens* SG				
*P. protegens*	*P. fluorescens*	27a	1	1
*P. saponiphila*	*P. fluorescens*	27a	2	2
New PS-6	*P. fluorescens*	27a, 27e	17	22
*P. fragi* SG				
*P. bubulae*	*P. fluorescens*	28a	2	8
*P. helleri*	*P. fluorescens*	31	1	1
*P. lundensis*	*P. fluorescens*	30	1	1
*P. weihenstephanensis*	*P. fluorescens*	29	1	2
*P. syringae* G				
New PS-7	*P. fluorescens*	36	1	1
New PS-8	*P. fluorescens*	36	1	1
*P. putida*G				
*P. alloputida*	*P. putida*	10b	12	88
*P. asiatica*	*P. putida*	10d	2	2
*P. capeferrum*	*P. putida*	8b	2	9
*P. entomophila*	*P. putida*	4, 5b	3	7
*P. fulva*	*P. putida*	12	4	21
*P. inefficax*	*P. putida*	10d	6	6
*P. juntendi*	*P. putida*	15	2	4
*P. putida*	*P. putida*	10a	1	1
*P. monteilii*	*P. putida*	10c	6	20
*P. mosselii*	*P. putida*	48, 25, 5b	4	4
*P. soli*	*P. putida*	5a, 5b	12	34
*P. uticensis*	*P. chlororaphis*	35b	1	2
P-GS1 (*P. putida* GB-1)	*P. putida*	14	1	1
P-GS2 (*Pseudomonas* sp. NBRC 111134)	*P. putida*	10a, 26	7	7
P-GS5 (*P. monteilii* GTC 10897)	*P. putida*	18	1	1
P-GS8 (*P. putida* PC2)	*P. putida*	23	1	1
P-GS10 (*P. putida* W619)	*P. putida*	8a, 8d, 9 (*n* = 1)	11	50
P-GS11 (*P. monteilii* USDA-ARS-USMARC-56711)	*P. putida*	40	1	1
P-GS13 (*Pseudomonas* sp. NBRC 111117)	*P. fluorescens*	35a, 28b	3	4
New PS-9	*P. putida*	11	2	2
New PS-10	*P. putida*	10e	1	1
New PS-11	*P. putida*	24	1	1
New PS-12	*P. putida*	16	1	1
New PS-13	*P. putida*	8c	2	7
New PS-14	*P. putida*	6	1	1
New PS-15	*P. putida*	22	1	1
New PS-16	*P. putida*	5c	5	13
New PS-17	*P. putida*	5b	6	8
New PS-18	*P. putida*	21	2	2
New PS-19	*P. fluorescens*	34	1	1
New PS-20	*P. viridiflava*	22	1	1
New PS-21	*P. oleovorans*	38	1	1
New PS-22	*P. viridiflava*	37	2	3
*P. oleovorans* G				
*P. guguanensis*	*P. mendocina*	41	1	1
*P. oleovorans/indoloxydans*	*P. oleovorans*	41	6	7
*P. toyotomiensis/chengduensis*	*P. mendocina*	41, 42	4	4
New PS-23	*P. oleovorans*	41	3	3
New PS-24	*P. mendocina*	43	1	1
*P. stutzeri* G				
*P. stutzeri* gv1	*P. stutzeri*	1	5	6
*P. stutzeri* gv3	*P. stutzeri*	2	1	1
*P. resinovorans* G				
*P. resinovorans*	*P. aeruginosa*	49	1	1
*P. aeruginosa* G				
*P. citronellolis*	*P. aeruginosa*	50	1	1
New PS-25	*P. alcaligenes*	44	1	1

*On the basis of the rpoD sequences, several groups are subdivided and labeled with letters (a, b, and c, etc.). ^a^Subgroups are indicated by letters.*

### Strains Identification Based on the *rpoD* Nucleotide Partial Sequence

**FIGURE 2:**
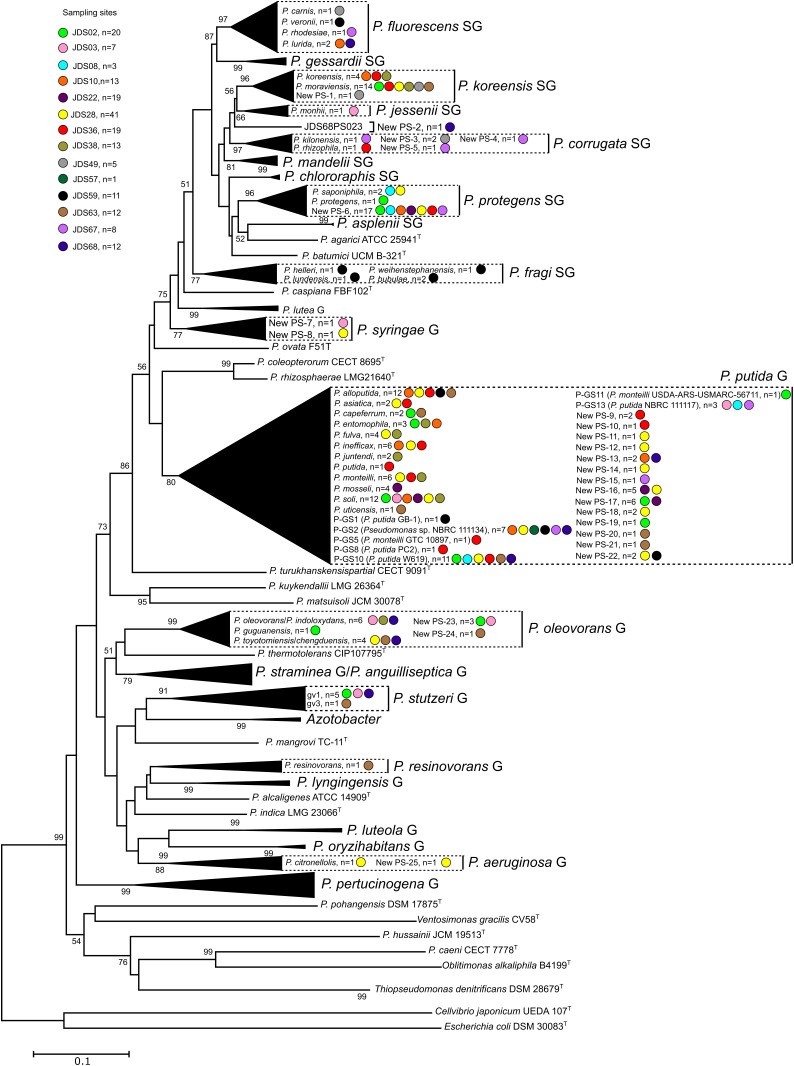
Phylogenetic relationships of the 190 selected strains based on the partial *rpoD* gene sequence analysis. Colored dots indicate the sampling site and “n” the number of strains. Distance matrices were calculated by the Jukes-Cantor evolutionary model (Jukes and Cantor, 1969). Dendrograms were generated by the neighbor-joining method. *Cellvibrio japonicum* UEDA 107^*T*^ was used as the outgroup. The bar indicates sequence divergence. Percentage bootstrap values of more than 50% (from 1000 replicates) are indicated at the nodes.

**FIGURE 3:**
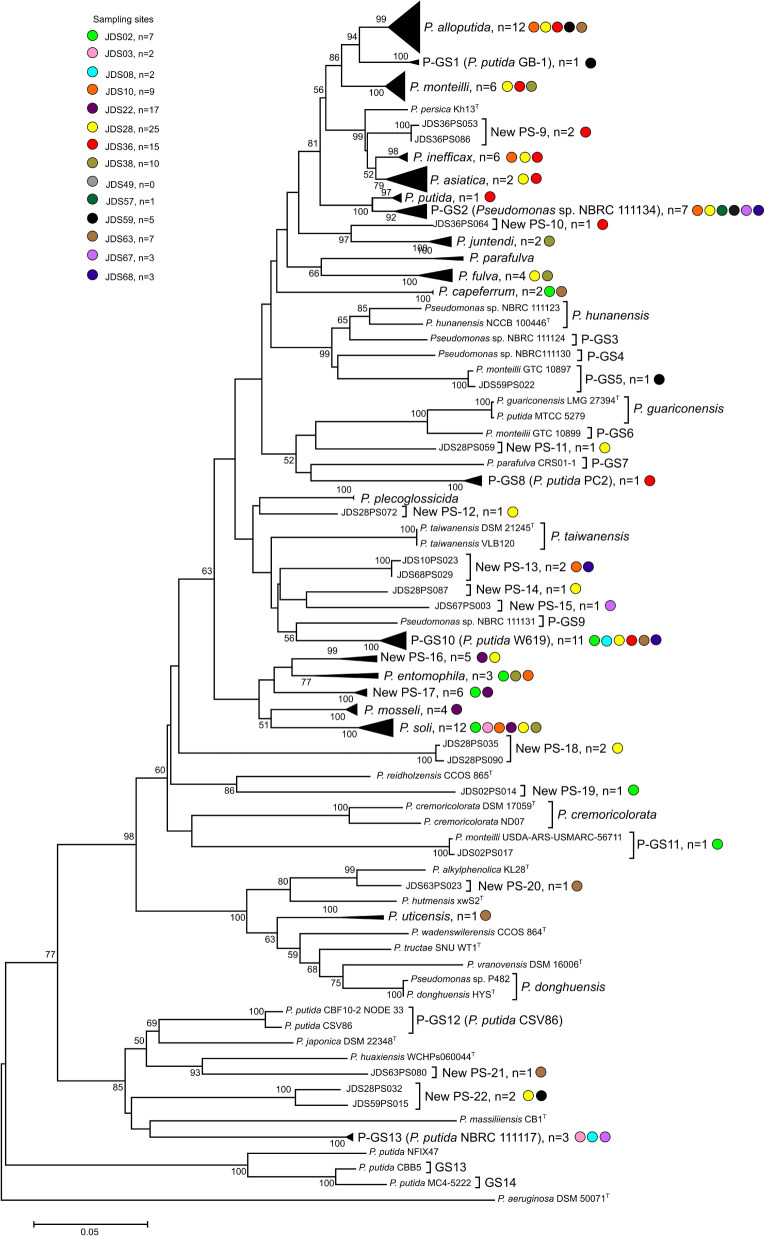
Phylogenetic tree of the strains in the *P. putida* phylogenetic group based on the partial *rpoD* gene sequence analysis. Colored dots indicate the sampling site and “n” the number of strains. Distance matrices were calculated by the Jukes-Cantor evolutionary model. Dendrograms were generated by the neighbor-joining method. *P. aeruginosa* ATCC 10145^*T*^ was used as the outgroup. The bar indicates sequence divergence. Percentage bootstrap values of more than 50% (from 1000 replicates) are indicated at the nodes.

Hundred ninety strains were selected as representatives of each MALDI-TOF MS cluster for their *rpoD* gene nucleotide sequence analysis to assign each grouping to a *Pseudomonas* species, as depicted in Figure 1 and Supplementary Figure S1 and Supplementary Table S2. For each of the 53 groupings at least two representative strains were selected when possible and are indicated in the Supplementary Figure S1 and Supplementary Table S2. The partial *rpoD* gene sequence of 190 strains was determined (Supplementary Table S2) and, as described in Materials and Methods, sequences were aligned together with an in house *rpoD* gene sequence database which contained 220 sequences of *Pseudomona*s type strains. Data obtained are shown in Table 1 and Supplementary Figure S1. Phylogenetic relationships are represented in Figures 2, 3 and Supplementary Figures S2, S3. Strains were identified at the species level when the similarity values with a species type strain was higher than 95%, the established species cutoff.

Following the phylogenetic classification proposed by Mulet et al. (2010) and Gomila et al. (2015), 108 strains (57%) affiliated to 34 phylospecies that were assigned to *Pseudomonas* known species. They were mainly distributed in the *P. fluorescens* lineage (Figure 2), in the *P. fluorescens* group (G), in six different phylogenetic subgroups of species (SG): *P. fluorescens* SG (four species), *P. fragi* SG (four species), *P. koreensis* SG (two species), *P. protegens* (two species), *P. corrugata* SG (two species) and *P. jessenii* (one species); 55 strains affiliated with the *P. putida* phylogenetic group of species (Figure 3) and were distributed in 12 known species; 19 strains affiliated to known species in the *P. aeruginosa* lineage, in three different groups: six in the *P. stutzeri* G (in genomovars 1 and 3), eleven in the *P. oleovorans* G (in three species) and two in the *P. aeruginosa* G (*P. resinovorans* and *P. citronellolis*) (Figure 2).

The remaining 82 strains (43% of the isolates sequenced) could not be assigned to any known *Pseudomonas* species, because their *rpoD* sequence similarities were below the cutoff value 95%, and were considered representatives of 32 putative new phylospecies. The putative new species were designated “New PS” followed by the corresponding number. When the whole genome sequence of the reference strain was known, it was designated “New GS” (GS for genomospecies) and the number of the reference strain was indicated in brackets. Strains of the putative new species were located in the *P. fluorescens* lineage, in the *P. fluorescens* G (*P. koreensis* SG, *P. corrugata* SG, and *P. protegens* SG), in the *P. syringae* G, and in the *P. putida* G. Strains in the *P. aeruginosa* lineage were distributed in the *P. aeruginosa* and *P. oleovorans* groups (Figures 1–3 and Table 1). Several groups or subgroups contained known and putative new species: *P. protegens* SG, *P. koreensis* G, *P. corrugata* SG, *P. aeruginosa* and *P. putida* G, *P. oleovorans* G.

Combining the identification based on the *rpoD* sequence and the groupings of strains in the MALDI-TOF MS analysis, we were able to classify 400 strains: 262 of them were assigned to known species, 65 to phylospecies for which a representative strain is well known, and 73 strains were assigned to new phylospecies not yet described. For instance, 31 of the 405 strains identified as *P. putida* with the VITEK MS system clustered in group 5a of the MALDI-TOF dendrogram. The *rpoD* gene of nine representative strains of this group were sequenced and showed 95–99.5 similarity with the corresponding sequence of the *P. soli* type strain, a member of the *P. putida* phylogenetic group; therefore, the 31 strains were identified as *P. soli* (Supplementary Figure S1). However, we have to consider that three other strains in the MALDI-TOF groups 5b, 7, and 51 were also identified by the *rpoD* sequence as *P. soli*. To verify the protein profiles, the 12 *P. soli* strains were reanalyzed in a Bruker autoflex III mass spectrometer and the Biotyper database as described in Peña et al. (2016). All strains clustered together with the *P. soli* type strain and were separated from the other species type strains in the corresponding dendrogram (results not shown).

The most abundant known phylospecies identified were: 88 strains of *P. alloputida*, 34 strains of *P. soli*, 20 strains of *P. monteilii*, 21 strains of *P. fulva* and 9 strains of *P. capeferrum* in the *P. putida* G; 14 strains of *P. moraviensi*s in the *P. koreensis* SG and 8 strains of *P. bubulae* in the *P. fragi* SG, both in the *P. fluorescens* G. *P stutzeri* was the eighth more abundant species with 7 strains, 6 of them assigned to genomovar 1, and 1 to genomovar 3 (Supplementary Table S1). From the 34 known phylospecies, 18 are represented by only 1 or 2 strains; the same situation is shown in the 32 new putative phylospecies because 23 of them are also represented by 1 or 2 strains.

The most abundant potential new phylospecies corresponded to species described in the *P. putida* G. They have to be considered potential new species, because their *rpoD* sequence similarity was below the species cutoff stablished with any type strain. One of these new phylospecies (50 strains) was represented by a well-studied strain assigned to the species *P. putida*: strain W619. This result suggested that these strains have to be reclassified in a species different to *P. putida.*

One hundred and ninety-one strains were assigned to phylogenetic groups or subgroups, but not at the phylospecies level, because several phylospecies were detected in the same MALDI-TOF grouping. Only three strains were identified in genera distinct to *Pseudomonas* and were located distantly in the dendrogram. One of them (branch 52) was identified as *Arthrobacter koreensis* and two (group 53) as *Brevundimonas terrae* by sequencing the 16S rDNA gene.

### Phylospecies Diversity and Statistical Indices

Rarefaction curves and phylospecies coverage indices were calculated for each sampling point and for the total set of strains identified to determine the extent of the species richness detected (Tables 2, 3). In total, 400 strains were identified at the phylospecies level (66% of the strains in the study) and were distributed in 65 phylospecies. The strains assigned to phylospecies ranged between 37 and 100% depending on the sampling point. The rarefaction curves did not reach saturation, indicating that the total diversity in the samples was not detected. Supplementary Figure S4 shows, as example, the results for five sampling sites (Supplementary Figure S4A) and for all the sampling sites (Supplementary Figure S4B). However, the species richness assessed with the coverage index per sampling point reached values between 80 and 94% in those points with a high number of isolates. Points with less than 32 strains showed coverages lower than 69%. The sampling sites with the highest richness were JDS28, JDS36, and JDS63, with 22, 18 and 15 phylospecies, respectively (Table 2). Seventy-eight strains of the 118 isolates from sampling site JDS28 were assigned to 22 phylospecies (Table 2). They showed a wide diversity of species, distributed in 6 different species of the *P. putida* G (17 *P. alloputida*, 11 *P. soli*, 3 *P. fulva*, 3 *P., monteilii*, 2 *P. inefficax*, and 1 *P. asiatica*), 3 species in *P. fluorescens* G (6 *P. moraviensis*, 1 *P. koreensis*, and 1 *P. saponiphila*), one species in the *P. aeruginosa* G (*P. citronellolis*), one species in the *P. oleovorans* G (1 *P. toyotomiensis*/*P. chengduensis*) and in 11 new phylospecies. The remaining 40 strains were assigned to four different phylogenetic groups.

**TABLE 2:** Strains assigned to species or to new phylospecies and their corresponding sampling sites of isolation.

Phylospecies	Number of sampling sites	JDS02	JDS03	JDS08	JDS10	JDS22	JDS28	JDS36	JDS38	JDS49	JDS57	JDS59	JDS63	JDS67	JDS68	Total	Group or subgroup
		**(nr isolates assignated to PS/nr isolates analised with rpoD gene)**															
*P. carnis*	2									1/1		1/0				2/1	*P. fluorescens* SG
*P. lurida*	2				1/1										2/1	3/2	*P. fluorescens* SG
*P. veronii*	1											1/1				1/1	*P. fluorescens* SG
*P. rhodesiae*	1													1/1		1/1	*P. fluorescens* SG
*P. koreensis*	3				2/2		1/1		1/1							4/4	*P. koreensis* SG
*P. moraviensis*	6	3/3					6/6	2/2	1/1	1/1			1/1			14/14	*P. koreensis* SG
New PS-1	1									1/1						1/1	*P. koreensis* SG
*P. mohnii*	1		1/1													1/1	*P. jessenii* SG
*P. kilonensis*	1													1/1		1/1	*P. corrugata* SG
*P. rhizophila*	1							1/1								1/1	*P. corrugata* SG
New PS-2	1														1/1	1/1	*P. corrugata* SG
New PS-3	1									2/2						2/2	*P. corrugata* SG
New PS-4	1													1/1		1/1	*P. corrugata* SG
New PS-5	1													1/1		1/1	*P. corrugata* SG
*P. protegens*	1	1/1														1/1	*P. protegens* SG
*P. saponiphila*	2			1/1			1/1									2/2	*P. protegens* SG
New PS-6	7	4/4		2/2	3/3	2/2	4/4	1/1						1/1		17/17	*P. protegens* SG
*P. bubulae*	2											7/2		1/0		8/2	*P. fragi* SG
*P. helleri*	1											1/1				1/1	*P. fragi* SG
*P. lundensis*	1											1/1				1/1	*P. fragi* SG
*P. weihenstephanensis*	1											2/1				2/1	*P. fragi* SG
New PS-7	1		1/1													1/1	*P. syringae* G
New PS-8	1						1/1									1/1	*P. syringae* G
*P. alloputida*	9				6/3		17/4	23/3	10/0		1	18/1	7/1	1/0	5/0	88/12	*P. putida* G
*P. asiatica*	2						1/1	1/1								2/2	*P. putida* G
*P. capeferrum*	3	1/1						3/0					5/1			9/2	*P. putida* G
*P. entomophila*	5	1/1			1/1	1/0		3/0	1/1							7/3	*P. putida* G
*P. fulva*	6						3/1	2/0	7/3	7/0				1/0	1/0	21/4	*P. putida* G
*P. inefficax*	3				2/2		2/2	2/2								6/6	*P. putida* G
*P. juntendi*	2								3/2			1/0				4/2	*P. putida* G
*P. putida*	1							1/1								1/1	*P. putida* G
*P. monteilii*	4						3/1	8/2	6/3			3/0				20/6	*P. putida* G
*P. mosselii*	1					4/4										4/4	*P. putida* G
*P. soli*	8	1/1	1/1		3/1	10/5	11/3	5/0	2/1				1/0			34/12	*P. putida* G
P-GS2 (*Pseudomonas* sp. NBRC 111134)	6				1/1		1/1				1/1	1/1		2/2	1/1	7/7	*P. putida* G
P-GS5 (*P. monteilii* GTC 10897)	1											1/1				1/1	*P. putida* G
P-GS8 (*P. putida* PC2)	1							1/1								1/1	*P. putida* G
P-GS10 (*P. putida* W619)	7	1/1		1/1			15/4	14/2	6/0				11/2		2/1	50/11	*P. putida* G
P-GS11 (*P. monteilii* USDA-ARS-USMARC-56711)	1	1/1														1/1	*P. putida* G
P-GS13 (*Pseudomonas* sp. NBRC 111117)	4		1/1	1/1									1/0	1/1		4/3	*P. putida* G
New PS-9	1							2/2								2/2	*P. putida* G
New PS-10	1							1/1								1/1	*P. putida* G
New PS-11	1						1/1									1/1	*P. putida* G
New PS-12	1						1/1									1/1	*P. putida* G
New PS-13	5				1/1				1/0	1/0			3/0		1/1	7/2	*P. putida* G
New PS-14	1						1/1									1/1	*P. putida* G
New PS-15	1													1/1		1/1	*P. putida* G
New PS-16	3					8/3	3/2	2/0								13/5	*P. putida* G
New PS-17	3	1/1			1/0	6/5										8/6	*P. putida* G
New PS-18	1						2/2									2/2	*P. putida* G
New PS-19	1	1/1														1/1	*P. putida* G
New PS-20	1												1/1			1/1	*P. putida* G
New PS-21	1												1/1			1/1	*P. putida* G
New PS-22	3						1/1					1/1	1/0			3/2	*P. putida* G
*P. guguanensis*	1	1/1														1/1	*P. oleovorans* G
*P. oleovorans/indoloxydans*	4		1/1					1/0	1/1						4/4	7/6	*P. oleovorans* G
*P. toyotomiensis/chengduensis*	3						1/1						1/1		2/2	4/4	*P. oleovorans* G
New PS-23	2	2/2	1/1													3/3	*P. oleovorans* G
New PS-24	1												1/1			1/1	*P. oleovorans* G
*P. stutzeri* gv1	3	3/3	2/1												1/1	6/5	*P. stutzeri* G
*P. stutzeri* gv3	1												1/1			1/1	*P. stutzeri* G
*P. resinovorans*	1												1/1			1/1	*P. aeruginosa* G
*P. citronellolis*	1						1/1									1/1	*P. aeruginosa* G
New PS-25	1						1/1									1/1	*P. aeruginosa* G
Phylospecies nr	-	13	7	4	10	6	22	18	11	6	2	13	15	11	10	-	

**TABLE 3:** Phylospecies richness and coverage index for each sampling site.

Sampling site	Number of strains	*rpoD* sequen-ced strains	Strains assigned to PS	%	Nr. of PS	Singl-etons	Cover-age (%)	Assignation to phylogenetic groups of strains not assigned to PS	
								Strains	Groups
JDS02	25	21	21	84	13	9	64	4	2
JDS03	8	7	8	100	7	6	25	0	0
JDS08	7	5	5	71	4	3	57	2	2
JDS10	32	15	21	66	10	5	84	11	4
JDS22	45	19	31	69	6	1	98	14	2
JDS28	118	41	78	66	22	12	90	40	5
JDS36	108	19	73	68	18	7	94	35	5
JDS38	45	13	39	87	11	5	89	6	3
JDS49	13	5	13	100	6	4	69	0	0
JDS57	2	1	2	100	2	2	0	0	0
JDS59	46	11	39	85	13	9	80	7	3
JDS63	102	12	38	37	15	10	90	64	5
JDS67	26	9	12	46	11	10	62	14	3
JDS68	34	12	20	59	10	5	85	14	4
Total	611	190	400	–	–	–	–	211	–

The rarefaction curve was also calculated for the whole course of the Danube River by considering the 400 strains assigned to phylospecies. Results indicate that 95% of the phylospecies diversity in the set of 611 strains was detected, but that a high *Pseudomonas* species richness present in the Danube River, at least 50% remains to be explored.

### Assignation to Species of Multidrug Resistant *Pseudomonas*

The previous data obtained for multidrug resistant *Pseudomonas* species (MDR) identified the strains in the species *P. putida* and *P. fluorescens* (Kittinger et al., 2016b). The *rpoD* sequence analysis, however, indicated that these assignations are correct at the level of phylogenetic group, *P. fluorescens* G, and *P. putida* G as defined by Mulet et al. (2010), but they can be now identified more precisely at the phylospecies level. The *P. fluorescens* group contains at the moment more than sixty different species and the *P. putida* group is composed of at least by 20 species (Keshavarz-Tohid et al., 2019; Peña et al., 2019). Table 4 shows the assignation by *rpoD* of the 14 MDR strains resistant to three or more antibiotic classes. They were assigned to seven phylospecies. Seven MDR strains were assigned to six known species, but the predominant phylospecies was New PS-6 with five isolates, that clustered in the MALDI-TOF dendrogram in group 27a composed by 45 strains. The closest species in the *rpoD* sequence is *P. protegens* in the *P. protegens* SG. New PS-6 strains could harbor the combination of resistances to three (MDR3) or four (MDR4) antibiotic classes and were distributed in three different sampling points: JDS02 (downstream Regensburg), JDS22 (downstream Budapest) and JDS28 (upstream Drava). The five strains of the New PS-6 are resistant to the antibiotics meropenem and ceftazidime, three of them also to imipenem, two of them to ciprofloxacin and the remaining three strains also to one more antibiotic (piperacilin/tazobactam, cefepime or gentamicin). These result indicated a broad spectrum of different combination of antibiotics resistances in the same phylospecies. Non-MDR strains of these seven phylospecies have been also isolated from the same or different sampling points. It has to be pointed out that, to our knowledge, none of the species detected have been found previously in clinical specimens, and therefore have to be considered environmental.

**TABLE 4:** Assignation to species or phylospecies of the MDR strains and their corresponding sampling sites of isolation.

Isolate	Phylospecies	Phylogenetic group	Resistance pattern^*a*^								MDR^*a*^	Location^*a*^
			TZP	CAZ	FEP	IMP	GM	MEM	CIP	LEV		
JDS02PS007	*P. protegens*	*P. fluorescens* G	S	R	S	S	S	R	R	S	MDR3	DS Regensburg, Germany
JDS02PS016	New PS-6	*P. fluorescens* G	R	R	S	S	R	R	R	S	MDR4	DS Regensburg, Germany
JDS02PS019	*P. soli*	*P. putida* G	R	S	S	S	S	R	R	S	MDR3	DS Regensburg, Germany
JDS02PS020	New PS-6	*P. fluorescens* G	S	R	S	S	S	R	R	S	MDR3	DS Regensburg, Germany
JDS22PS016	New PS-16	*P. putida* G	R	R	S	S	S	R	S	S	MDR3	DS Budapest, Hungary
JDS22PS018	New PS-6	*P. fluorescens* G	S	R	S	R	S	R	S	S	–	DS Budapest, Hungary
JDS22PS032	*P. soli*	*P. putida* G	R	S	S	R	S	R	S	S	–	DS Budapest, Hungary
JDS22PS035	New PS-16	*P. putida* G	R	S	S	R	S	R	S	S	–	DS Budapest, Hungary
JDS22PS043	*P. soli*	*P. putida* G	R	S	S	S	S	R	R	S	MDR3	DS Budapest, Hungary
JDS28PS083	New PS-6	*P. fluorescens* G	S	R	R	R	S	R	S	S	–	US Drava, Croatia/Serbia
JDS28PS113	New PS-6	*P. fluorescens* G	S	R	S	R	S	R	S	S	–	US Drava, Croatia/Serbia
JDS28PS115	*P. inefficax*	*P. putida* G	R	S	S	S	S	R	R	R	MDR3	US Drava, Croatia/Serbia
JDS28PS117	*P. asiatica*	*P. putida* G	R	S	S	S	S	R	S	R	MDR3	US Drava, Croatia/Serbia
JDS59PS020	*P. bubulae*	*P. fluorescens* G	R	R	R	S	S	S	S	S	–	DS Arges, Romania/Bulgaria

*^a^Data obtained from Kittinger et al. (2016a). Multidrug resistance was assigned to an isolate if it revealed a resistance to three (MDR 3) or four (MDR 4) antibiotic classes. Antibiotic classes: acylureidopenicillins: piperacilin/tazobactam (TZP); cephalosporins: ceftazidime (CAZ), cefepime (FEP); carbapenems: meropenem (MEM), imipenem (IMP); aminoglycosides: gentamicin (GM); fluoroquinolones: ciprofloxacin (CIP), levofloxacin (LEV).*

## Discussion

The utility of the partial *rpoD* gene sequence for identification and phylogenetic studies in the genus *Pseudomonas* was first proposed by Yamamoto et al. (2000), and later confirmed by Mulet et al. (2010) as well as by Ghyselinck et al. (2013) and Scotta et al. (2013). The sequence of this housekeeping gene has been used not only for the description of new environmental *Pseudomonas* species from various aquatic habitats, like rivers (Sánchez et al., 2014) or intertidal seashore (Mulet et al., 2011) but also for clinical strains (Scotta et al., 2013) or plants (Beiki et al., 2016). The good concordance in the *Pseudomonas* genus between the protein profiles obtained by MALDI-TOF MS and the *rpoD* gene nucleotide sequence was also demonstrated (Mulet et al., 2012), but it is generally accepted that the *rpoD* gene sequence comparison is a better approach for the species differentiation in the *Pseudomonas* genus. This assumption is also demonstrated in the present work. Two hundred strains remain to be identified at the species level by *rpoD* sequence combined with protein profile, but were classified at the phylogenetic group or subgroup levels. In the strains collection studied, we do not expect a significant increase in the number of *Pseudomonas* species detected by increasing the sequencing effort of the isolates, because most of these 200 strains were isolated from samples well characterized statistically, with phylospecies coverage indices higher than 80%. However, we have to point out that the set of 611 strains was isolated at 37°C, a temperature not frequently used for the isolation of environmental *Pseudomonas*. Although the majority of *Pseudomonas* can grow at 37°C, a significant percentage of species could not have been isolated and, therefore, it was not detected in the present study and we conclude that the diversity must be even greater than that found.

### MALDI-TOF vs. *rpoD* Sequence Identifications

The VITEK MS identification system clearly assigned the strains to the correct phylogenetic group of species, but failed at the species level in many strains. Forty-one strains were assigned by MALDI-TOF to a species different from the identification obtained by the combined MALDI-TOF/***rpoD*** sequencing procedure. Additionally, other 42 strains have to be considered possible new species under a phylogenetic point of view. This significant discrepancy is probably due to the extent of the VITEK MS database, designed mainly for the identification of clinically relevant species and has to be updated with environmental species, not included in the commercial database. Other authors, even in the clinical laboratory (McElvania TeKippe and Burnham, 2014), have also addressed the difficulties of the MALDI-TOF MS system for the identification of species in the ***P. putida*** phylogenetic group. However, a fair good concordance was found in the assignation of strains in the 53 MALDI-TOF groupings and the corresponding phylogenetic group or subgroup. Three possible situations were detected:

(i) all strains in a MALDI-TOF group corresponded to a single phylospecies, that indicates a perfect match between both methods; this is the situation detected in *P. stutzeri* strains, that were correctly identified by both methods. The protein profiles allowed even the differentiation of genomovar 1 and 3 within the species.
(ii) a single MALDI-TOF subgroup corresponded to more than one phylospecies, that indicates a better resolution of the *rpoD* sequence over the protein profile; VITEK MS was not able to differentiate the 27 phylospecies in the *P. putida* phylogenetic group and identified the strains as *P. putida*. Moreover, the *rpoD* sequence revealed the existence of phylospecies not yet described. Strains in MALDI-TOF group 28 were identified as *P. fluorescens* or *P. chlororaphis* by the VITEK MS identification system, but *rpoD* sequences demonstrate that this group included strains of the species *P. koreensis*, *P. moraviensis*, and 1 other possible new phylospecies. All of them belong to the phylogenetic SG of *P. koreensis*, within the *P. fluorescens* G.
(iii) in few cases strains of the same phylospecies were scattered in different MALDI-TOF groups; e.g., the presence of three *P. soli* strains in groups 5B, 7, and 51. This fact can be attributed to technical errors in obtaining the mass spectra, to errors in the data processing or the low discriminatory power of the MALDI-TOF MS technique to differentiate strains of closely related species.

At least 73 strains were classified in 25 phylospecies of not yet described bacterial species (labeled New-PS) because their *rpoD* gene sequences were less than 95% similar to species type strains in the genus: 14 in the *P. putida*, 6 in the *P. fluorescens*, 2 in the *P. syringae*, 2 in the *P. oleovorans* and 1 in the *P. aeruginosa* phylogenetic groups. These possible new species merit deeply taxonomic studies.

We analyzed in a previous publication the diversity of *Pseudomonas* in the Woluve River by analyzing the *rpoD* sequences from cultivated strains and by pyrosequencing *rpoD* amplicons from total DNA extracted from water samples (Sánchez et al., 2014). Two hundred and forty-six possible new phylospecies were detected in the amplicon analyses and were not found in the cultivated strains. Interestingly, two Danube River strains are 97% identical to two of the 246 putative novel phylospecies detected in the Woluve River by pyrosequencing an *rpoD* amplicon: strains JDS49PS004 (New PS-1) and JDS02PS014 (New PS-19), confirming in this way the value of the *rpoD* sequences to detect *Pseudomonas* diversity and predict new species by culture independent methods.

### Antibiotic Resistances

Correct and precise species identification is also a prerequisite for the assessment of antimicrobial susceptibilities of the isolates, because the intrinsic, or basal, resistance will vary significantly among species and even different strains. MALDI-TOF identified the 14 MDR strains as members of the species *P. putida* or *P. fluorescens*. However, the *rpoD* sequence analysis demonstrate that they belong to seven different *Pseudomonas* species, five known (*P. protegens*, *P. soli, P. inefficax, P. asiatica*, and *P. bubulae*) and two other possible new species (PS-6 and PS-16) all in the *P. fluorescens* or *P. putida* phylogenetic groups of species. For all of them, non-MDR strains were also detected, which suggests a process of resistance acquisition. These strains belong to species not described as pathogens and represent an environmental reservoir of antibiotic resistance genes that can be transferred to other species. Other authors have found a MDR strain of *P. aeruginosa* (opportunistic pathogen) in a water sample of the Woluve River (Belgium) (Pirnay et al., 2005) but also indicating that the majority of the Woluve strains were antibiotics susceptible. It is worth to mention, that we did not find any *P. aeruginosa* strain in the Danube River isolates, an abundant species in aquatic environments and considered indicator for water risk assessments (Mena and Gerba, 2009).

## Conclusion

We can conclude that the assignation at the genus and at the phylogenetic group or subgroup levels obtained by MALDI-TOF MS is accurate, but lacks sufficient resolution at the species level in the genus *Pseudomonas*. This accuracy can be provided by *rpoD* sequence analyses. MALDI-TOF MS is a good method for dereplication of large numbers of isolates. Additionally, the discovery of a high percentage of 32 possible new phylospecies indicate that the number of species in the genus is much higher than the currently known, more than 220 species. One of this possible new phylospecies was previously detected by culture-independent methods also in a river sample. This result emphasizes the value of *rpoD* sequences at species-level identification and for predicting new species in the *Pseudomonas* genus by culture-independent analyses. The potential new species found are further analyzed taxonomically to propose them as new species. We also demonstrate that the “culturomics” strategy based on classical culture techniques and exhaustive analyses of isolates is a simple and valuable tool to study bacterial diversity. One of the most significant effect of this approach has been the rapid increase in the number of bacterial species described with validly published names (Abdallah et al., 2017).

## Data Availability Statement

The datasets presented in this study can be found in online repositories. The names of the repository/repositories and accession number(s) can be found in the article/Supplementary Material.

## Author Contributions

CK, GZ, JL, and EG-V designed the project and analyzed the data. MMu, MMo, DR, and MG performed the experiments. All authors reviewed and approved the manuscript.

## Conflict of Interest

The authors declare that the research was conducted in the absence of any commercial or financial relationships that could be construed as a potential conflict of interest.

## Supplementary Material

FIGURE S1 MALDI-TOF MS dendrogram of the 611 *Pseudomonas* strains studied, their origin and assignation to MALDI-TOF groupings and phylospecies.

FIGURE S2 Phylogenetic relationships of the selected strains based on the partial *rpoD* gene sequence analysis. Distance matrices were calculated by the Jukes-Cantor method. Dendrograms were generated by the neighbor-joining method. *Cellvibrio japonicum* UEDA 107^*T*^ was used as the outgroup. The bar indicates sequence divergence. Percentage bootstrap values of more than 50% (from 1000 replicates) are indicated at the nodes. Filled circles indicate that the corresponding nodes were also obtained in the trees generated with the maximum-likelihood and maximum parsimony methods.

FIGURE S3 Phylogenetic tree of the strains in the *P. putida* phylogenetic group based on the partial *rpoD* gene sequence analysis. Distance matrices were calculated by the Jukes-Cantor method. Dendrograms were generated by the neighbor-joining method. *P. aeruginosa* ATCC 10145^*T*^ was used as the outgroup. The bar indicates sequence divergence. Percentage bootstrap values of more than 50% (from 1000 replicates) are indicated at the nodes. Filled circles indicate that the corresponding nodes were also obtained in the trees generated with the maximum-likelihood and maximum parsimony methods.

FIGURE S4 Rarefaction curves. **(A)** Individual curves for the five sampling sites with the highest numbers of isolates; **(B)** All sampling sites and isolates.

TABLE S1 Location of the 14 sampling points along the Danube River and date of isolation.

TABLE S2 Partial *rpoD* gene sequences obtained in the present study and their corresponding accession numbers.
