# Supplementary material for: Pseudomonas Species Diversity Along the Danube River Assessed by rpoD Gene Sequence and MALDI-TOF MS Analyses of Cultivated Strains
Source: Front Microbiol. 2020 Sep 2;11:2114. doi: 10.3389/fmicb.2020.02114 (PMC7492575; doi:10.3389/fmicb.2020.02114)
Supplement: FIGURE S1 — MALDI-TOF MS dendrogram of the 611 Pseudomonas strains studied, their origin and assignation to MALDI-TOF groupings and phylospecies. [file Image_1.pdf]

FIGURE S1. MALDI-TOF MS dendrogram of the 611 *Pseudomonas* strains studied, their origin and assignation to MALDI-TOF groupings and phylospecies.

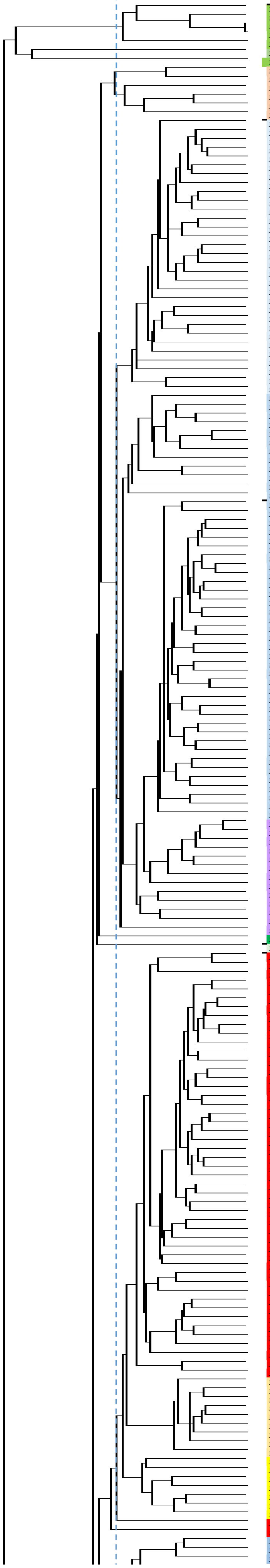

Isolate	WC-MALDI-TOF MS (VITEK) identification	WC-MALDI-TOF MS Group/Subgroup		Assignation to Phylospecies	[a]Assignation to Phylospecies with *rpoD* gene	Group/Subgroup
JDS02PS001	*P. stutzeri*	1	1	*P. stutzeri* gvI	*P. stutzeri* gvI	*P. stutzeri* G
JDS02PS004	*P. stutzeri*	1	1	*P. stutzeri* gvI	*P. stutzeri* gvI	*P. stutzeri* G
JDS03PS002	*P. stutzeri*	1	1	*P. stutzeri* gvI	*P. stutzeri* gvI	*P. stutzeri* G
JDS03PS003	*P. stutzeri*	1	1	*P. stutzeri* gvI		*P. stutzeri* G
JDS02PS008	*P. stutzeri*	1	1	*P. stutzeri* gvI	*P. stutzeri* gvI	*P. stutzeri* G
JDS63PS033	*P. stutzeri*	2	2	*P. stutzeri* gv3	*P. stutzeri* gv3	*P. stutzeri* G
JDS68PS016	*P. stutzeri*	3	3	*P. stutzeri* gvI	*P. stutzeri* gvI	*P. stutzeri* G
JDS02PS002	*P. putida*	4	4	*P. entomophila*	*P. entomophila*	*P. putida* G
JDS22PS001	*P. putida*	4	4	*P. entomophila*		*P. putida* G
JDS36PS003	*P. putida*	4	4	*P. entomophila*		*P. putida* G
JDS36PS046	*P. putida*	4	4	*P. entomophila*		*P. putida* G
JDS36PS073	*P. putida*	4	4	*P. entomophila*		*P. putida* G
JDS38PS018	*P. putida*	4	4	*P. entomophila*	*P. entomophila*	*P. putida* G
JDS02PS019	*P. putida*	5	5a	*P. soli*	*P. soli*	*P. putida* G
JDS03PS001	*P. putida*	5	5a	*P. soli*	*P. soli*	*P. putida* G
JDS28PS019	*P. putida*	5	5a	*P. soli*		*P. putida* G
JDS28PS034	*P. putida*	5	5a	*P. soli*		*P. putida* G
JDS28PS038	*P. putida*	5	5a	*P. soli*		*P. putida* G
JDS10PS009	*P. putida*	5	5a	*P. soli*		*P. putida* G
JDS36PS105	*P. putida*	5	5a	*P. soli*		*P. putida* G
JDS28PS049	*P. putida*	5	5a	*P. soli*		*P. putida* G
JDS22PS025	*P. putida*	5	5a	*P. soli*	*P. soli*	*P. putida* G
JDS22PS043	*P. putida*	5	5a	*P. soli*	*P. soli*	*P. putida* G
JDS22PS044	*P. putida*	5	5a	*P. soli*	*P. soli*	*P. putida* G
JDS22PS022	*P. putida*	5	5a	*P. soli*		*P. putida* G
JDS22PS046	*P. putida*	5	5a	*P. soli*		*P. putida* G
JDS28PS010	*P. putida*	5	5a	*P. soli*		*P. putida* G
JDS10PS006	*P. putida*	5	5a	*P. soli*		*P. putida* G
JDS36PS030	*P. putida*	5	5a	*P. soli*		*P. putida* G
JDS28PS112	*P. putida*	5	5a	*P. soli*		*P. putida* G
JDS28PS091	*P. putida*	5	5a	*P. soli*	*P. soli*	*P. putida* G
JDS63PS100	*P. putida*	5	5a	*P. soli*		*P. putida* G
JDS28PS026	*P. putida*	5	5a	*P. soli*		*P. putida* G
JDS22PS017	*P. putida*	5	5a	*P. soli*		*P. putida* G
JDS22PS024	*P. putida*	5	5a	*P. soli*		*P. putida* G
JDS36PS014	*P. putida*	5	5a	*P. soli*		*P. putida* G
JDS22PS019	*P. putida*	5	5a	*P. soli*		*P. putida* G
JDS22PS028	*P. putida*	5	5a	*P. soli*	*P. soli*	*P. putida* G
JDS36PS055	*P. putida*	5	5a	*P. soli*		*P. putida* G
JDS28PS108	*P. putida*	5	5a	*P. soli*		*P. putida* G
JDS28PS103	*P. putida*	5	5a	*P. soli*	*P. soli*	*P. putida* G
JDS38PS030	*P. putida*	5	5a	*P. soli*		*P. putida* G
JDS28PS030	*P. putida*	5	5a	*P. soli*	*P. soli*	*P. putida* G
JDS36PS063	*P. putida*	5	5a	*P. soli*		*P. putida* G
JDS02PS021	*P. putida*	5	5b	New PS-17	New PS-17	*P. putida* G
JDS10PS019	*P. putida*	5	5b	New PS-17		*P. putida* G
JDS22PS031	*P. putida*	5	5b	New PS-17	New PS-17	*P. putida* G
JDS22PS036	*P. putida*	5	5b	New PS-17	New PS-17	*P. putida* G
JDS22PS023	*P. putida*	5	5b	New PS-17		*P. putida* G
JDS22PS029	*P. putida*	5	5b	New PS-17	New PS-17	*P. putida* G
JDS28PS006	*P. putida*	5	5b	*P. entomophila*, *P. mosselii*, New PS-17		*P. putida* G
JDS68PS020	*P. putida*	5	5b	*P. entomophila*, *P. mosselii*, New PS-17		*P. putida* G
JDS10PS030	*P. fluorescens*	5	5b	*P. entomophila*, *P. mosselii*, New PS-17		*P. putida* G
JDS36PS072	*P. putida*	5	5b	*P. entomophila*, *P. mosselii*, New PS-17		*P. putida* G
JDS22PS039	*P. putida*	5	5b	New PS-17	New PS-17	*P. putida* G
JDS36PS047	*P. putida*	5	5b	*P. entomophila*, *P. mosselii*, New PS-17		*P. putida* G
JDS10PS003	*P. putida*	5	5b	*P. entomophila*	*P. entomophila*	*P. putida* G
JDS22PS045	*P. putida*	5	5b	*P. entomophila*, *P. mosselii*, New PS-17		*P. putida* G
JDS10PS013	*P. putida*	5	5b	*P. entomophila*, *P. mosselii*, New PS-17		*P. putida* G
JDS28PS025	*P. putida*	5	5b	*P. entomophila*, *P. mosselii*, New PS-17		*P. putida* G
JDS36PS109	*P. putida*	5	5b	*P. entomophila*, *P. mosselii*, New PS-17		*P. putida* G
JDS63PS014	*P. putida*	5	5b	*P. entomophila*, *P. mosselii*, New PS-17		*P. putida* G
JDS22PS026	*P. putida*	5	5b	New PS-17	New PS-17	*P. putida* G
JDS28PS015	*P. putida*	5	5b	*P. entomophila*, *P. mosselii*, New PS-17		*P. putida* G
JDS36PS002	*P. putida*	5	5b	*P. entomophila*, *P. mosselii*, New PS-17		*P. putida* G
JDS22PS014	*P. putida*	5	5b	*P. entomophila*, *P. mosselii*, New PS-17		*P. putida* G
JDS36PS083	*P. putida*	5	5b	*P. entomophila*, *P. mosselii*, New PS-17		*P. putida* G
JDS22PS030	*P. putida*	5	5b	*P. entomophila*, *P. mosselii*, New PS-17		*P. putida* G
JDS36PS034	*P. putida*	5	5b	*P. entomophila*, *P. mosselii*, New PS-17		*P. putida* G
JDS28PS054	*P. putida*	5	5b	*P. entomophila*, *P. mosselii*, New PS-17		*P. putida* G
JDS28PS029	*P. putida*	5	5b	*P. entomophila*, *P. mosselii*, New PS-17		*P. putida* G
JDS28PS037	*P. putida*	5	5b	*P. entomophila*, *P. mosselii*, New PS-17		*P. putida* G
JDS22PS033	*P. putida*	5	5b	*P. entomophila*, *P. mosselii*, New PS-17		*P. putida* G
JDS28PS036	*P. putida*	5	5b	*P. entomophila*, *P. mosselii*, New PS-17		*P. putida* G
JDS22PS041	*P. putida*	5	5b	*P. entomophila*, *P. mosselii*, New PS-17		*P. putida* G
JDS22PS042	*P. putida*	5	5b	*P. mosselii*	*P. mosselii*	*P. putida* G
JDS28PS022	*P. putida*	5	5b	*P. entomophila*, *P. mosselii*, New PS-17		*P. putida* G
JDS36PS062	*P. putida*	5	5b	*P. entomophila*, *P. mosselii*, New PS-17		*P. putida* G
JDS22PS003	*P. putida*	5	5b	*P. entomophila*, *P. mosselii*, New PS-17		*P. putida* G
JDS22PS020	*P. putida*	5	5b	*P. entomophila*, *P. mosselii*, New PS-17		*P. putida* G
JDS63PS093	*P. putida*	5	5b	*P. entomophila*, *P. mosselii*, New PS-17		*P. putida* G
JDS28PS007	*P. putida*	5	5b	*P. entomophila*, *P. mosselii*, New PS-17		*P. putida* G
JDS28PS044	*P. putida*	5	5b	*P. entomophila*, *P. mosselii*, New PS-17		*P. putida* G
JDS28PS110	*P. putida*	5	5b	*P. entomophila*, *P. mosselii*, New PS-17		*P. putida* G
JDS36PS035	*P. putida*	5	5b	*P. entomophila*, *P. mosselii*, New PS-17		*P. putida* G
JDS22PS032	*P. putida*	5	5b	*P. soli*	*P. soli*	*P. putida* G
JDS22PS037	*P. putida*	5	5b	*P. mosselii*	*P. mosselii*	*P. putida* G
JDS28PS045	*P. putida*	5	5b	*P. entomophila*, *P. mosselii*, New PS-17		*P. putida* G
JDS28PS069	*P. putida*	5	5b	*P. entomophila*, *P. mosselii*, New PS-17		*P. putida* G
JDS22PS021	*P. putida*	5	5b	*P. entomophila*, *P. mosselii*, New PS-17		*P. putida* G
JDS36PS029	*P. putida*	5	5b	*P. entomophila*, *P. mosselii*, New PS-17		*P. putida* G
JDS22PS015	*P. putida*	5	5b	*P. entomophila*, *P. mosselii*, New PS-17		*P. putida* G
JDS22PS004	*P. putida*	5	5c	New PS-16	New PS-16	*P. putida* G
JDS22PS009	*P. putida*	5	5c	New PS-16		*P. putida* G
JDS22PS016	*P. putida*	5	5c	New PS-16	New PS-16	*P. putida* G
JDS22PS008	*P. putida*	5	5c	New PS-16		*P. putida* G
JDS22PS038	*P. putida*	5	5c	New PS-16		*P. putida* G
JDS36PS085	*P. putida*	5	5c	New PS-16		*P. putida* G
JDS22PS035	*P. putida*	5	5c	New PS-16	New PS-16	*P. putida* G
JDS28PS096	*P. putida*	5	5c	New PS-16	New PS-16	*P. putida* G
JDS22PS027	*P. putida*	5	5c	New PS-16		*P. putida* G
JDS22PS040	*P. putida*	5	5c	New PS-16		*P. putida* G
JDS28PS111	*P. putida*	5	5c	New PS-16		*P. putida* G
JDS36PS070	*P. putida*	5	5c	New PS-16		*P. putida* G
JDS28PS080	*P. putida*	5	5c	New PS-16	New PS-16	*P. putida* G
JDS28PS087	*P. putida*	6	6	New PS-14	New PS-14	*P. putida* G
JDS38PS027	*P. putida*	7	7	*P. soli*	*P. soli*	*P. putida* G
JDS02PS022	*P. putida*	8	8a	P-GS10 (*P. putida* W619)	P-GS10 (*P. putida* W619)	*P. putida* G
JDS28PS005	*P. putida*	8	8a	P-GS10 (*P. putida* W619)		*P. putida* G
JDS08PS006	*P. putida*	8	8a	P-GS10 (*P. putida* W619)	P-GS10 (*P. putida* W619)	*P. putida* G
JDS28PS016	*P. putida*	8	8a	P-GS10 (*P. putida* W619)		*P. putida* G
JDS68PS017	*P. putida*	8	8a	P-GS10 (*P. putida* W619)		*P. putida* G
JDS28PS042	*P. putida*	8	8a	P-GS10 (*P. putida* W619)		*P. putida* G
JDS36PS040	*P. mendocina*	8	8a	P-GS10 (*P. putida* W619)	P-GS10 (*P. putida* W619)	*P. putida* G
JDS38PS004	*P. putida*	8	8a	P-GS10 (*P. putida* W619)		*P. putida* G
JDS36PS081	*P. putida*	8	8a	P-GS10 (*P. putida* W619)		*P. putida* G
JDS36PS088	*P. putida*	8	8a	P-GS10 (*P. putida* W619)		*P. putida* G
JDS36PS082	*P. putida*	8	8a	P-GS10 (*P. putida* W619)		*P. putida* G
JDS36PS026	*P. putida*	8	8a	P-GS10 (*P. putida* W619)		*P. putida* G
JDS63PS099	*P. putida*	8	8a	P-GS10 (*P. putida* W619)		*P. putida* G
JDS28PS074	*P. putida*	8	8a	P-GS10 (*P. putida* W619)		*P. putida* G
JDS28PS086	*P. putida*	8	8a	P-GS10 (*P. putida* W619)	P-GS10 (*P. putida* W619)	*P. putida* G
JDS28PS079	*P. putida*	8	8a	P-GS10 (*P. putida* W619)	P-GS10 (*P. putida* W619)	*P. putida* G
JDS38PS039	*P. putida*	8	8a	P-GS10 (*P. putida* W619)		*P. putida* G
JDS63PS062	*P. putida*	8	8a	P-GS10 (*P. putida* W619)		*P. putida* G
JDS28PS040	*P. putida*	8	8a	P-GS10 (*P. putida* W619)		*P. putida* G
JDS38PS007	*P. putida*	8	8a	P-GS10 (*P. putida* W619)		*P. putida* G
JDS36PS043	*P. putida*	8	8a	P-GS10 (*P. putida* W619)		*P. putida* G
JDS28PS043	*P. putida*	8	8a	P-GS10 (*P. putida* W619)		*P. putida* G
JDS36PS011	*P. putida*	8	8a	P-GS10 (*P. putida* W619)		*P. putida* G
JDS36PS056	*P. putida*	8	8a	P-GS10 (*P. putida* W619)		*P. putida* G
JDS63PS040	*P. putida*	8	8a	P-GS10 (*P. putida* W619)		*P. putida* G
JDS36PS041	*P. putida*	8	8a	P-GS10 (*P. putida* W619)		*P. putida* G
JDS36PS005	*P. putida*	8	8a	P-GS10 (*P. putida* W619)		*P. putida* G
JDS36PS059	*P. putida*	8	8a	P-GS10 (*P. putida* W619)		*P. putida* G
JDS38PS024	*P. putida*	8	8a	P-GS10 (*P. putida* W619)		*P. putida* G
JDS38PS033	*P. putida*	8	8a	P-GS10 (*P. putida* W619)		*P. putida* G
JDS28PS061	*P. putida*	8	8a	P-GS10 (*P. putida* W619)		*P. putida* G
JDS63PS027	*P. putida*	8	8a	P-GS10 (*P. putida* W619)		*P. putida* G
JDS36PS042	*P. putida*	8	8a	P-GS10 (*P. putida* W619)		*P. putida* G
JDS36PS013	*P. putida*	8	8a	P-GS10 (*P. putida* W619)		*P. putida* G
JDS28PS116	*P. putida*	8	8a	P-GS10 (*P. putida* W619)		*P. putida* G
JDS68PS021	*P. putida*	8	8a	P-GS10 (*P. putida* W619)	P-GS10 (*P. putida* W619)	*P. putida* G
JDS28PS001	*P. putida*	8	8a	P-GS10 (*P. putida* W619)	P-GS10 (*P. putida* W619)	*P. putida* G
JDS63PS028	*P. putida*	8	8a	P-GS10 (*P. putida* W619)		*P. putida* G
JDS38PS031	*P. putida*	8	8a	P-GS10 (*P. putida* W619)		*P. putida* G
JDS28PS070	*P. putida*	8	8a	P-GS10 (*P. putida* W619)		*P. putida* G
JDS63PS053	*P. putida*	8	8a	P-GS10 (*P. putida* W619)		*P. putida* G
JDS63PS017	*P. putida*	8	8a	P-GS10 (*P. putida* W619)		*P. putida* G
JDS28PS109	*P. putida*	8	8a	P-GS10 (*P. putida* W619)		*P. putida* G
JDS63PS024	*P. putida*	8	8a	P-GS10 (*P. putida* W619)		*P. putida* G
JDS63PS004	*P. putida*	8	8a	P-GS10 (*P. putida* W619)		*P. putida* G
JDS36PS012	*P. putida*	8	8a	P-GS10 (*P. putida* W619)	P-GS10 (*P. putida* W619)	*P. putida* G
JDS28PS039	*P. putida*	8	8a	P-GS10 (*P. putida* W619)		*P. putida* G
JDS28PS041	*P. putida*	8	8a	P-GS10 (*P. putida* W619)	P-GS10 (*P. putida* W619)	*P. putida* G
JDS02PS023	*P. putida*	8	8b	*P. capeferrum*	*P. capeferrum*	*P. putida* G
JDS36PS100	*P. putida*	8	8b	*P. capeferrum*		*P. putida* G
JDS63PS041	*P. putida*	8	8b	*P. capeferrum*		*P. putida* G
JDS36PS103	*P. putida*	8	8b	*P. capeferrum*		*P. putida* G
JDS63PS038	*P. putida*	8	8b	*P. capeferrum*		*P. putida* G
JDS63PS085	*P. putida*	8	8b	*P. capeferrum*		*P. putida* G
JDS63PS066	*P. putida*	8	8b	*P. capeferrum*		*P. putida* G
JDS36PS099	*P. putida*	8	8b	*P. capeferrum*		*P. putida* G
JDS63PS016	*P. putida*	8	8b	*P. capeferrum*	*P. capeferrum*	*P. putida* G
JDS10PS023	*P. putida*	8	8c	New PS-13	New PS-13	*P. putida* G
JDS63PS054	*P. putida*	8	8c	New PS-13		*P. putida* G
JDS38PS019	*P. putida*	8	8c	New PS-13		*P. putida* G
JDS49PS002	*P. putida*	8	8c	New PS-13		*P. putida* G
JDS63PS050	*P. putida*	8	8c	New PS-13		*P. putida* G
JDS63PS094	*P. putida*	8	8c	New PS-13		*P. putida* G
JDS68PS029	*P. putida*	8	8c	New PS-13	New PS-13	*P. putida* G
JDS63PS068	*P. putida*	8	8d	P-GS10 (*P. putida* W619)	P-GS10 (*P. putida* W619)	*P. putida* G
JDS63PS026	*P. putida*	9	9	P-GS10 (*P. putida* W619)	P-GS10 (*P. putida* W619)	*P. putida* G
JDS08PS007	*P. putida*	10	10a	*P. putida*, P-GS2 (*Pseudomonas* sp. NBRC 111134)		*P. putida* G
JDS10PS028	*P. putida*	10	10a	*P. putida*, P-GS2 (*Pseudomonas* sp. NBRC 111134)		*P. putida* G
JDS10PS007	*P. putida*	10	10a	P-GS2 (*Pseudomonas* sp. NBRC 111134)	P-GS2 (*Pseudomonas* sp. NBRC 111134)	*P. putida* G

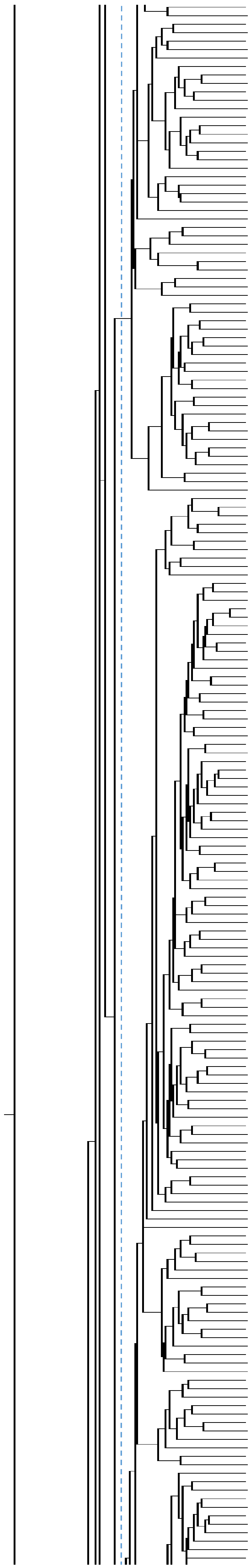

JDS63PS057	*P. putida*	10	10a	*P. putida* , P-GS2 (*Pseudomonas* sp. NBRC 111134)		*P. putida* G
JDS68PS009	*P. putida*	10	10a	*P. putida* , P-GS2 (*Pseudomonas* sp. NBRC 111134)		*P. putida* G
JDS28PS062	*P. putida*	10	10a	*P. putida* , P-GS2 (*Pseudomonas* sp. NBRC 111134)		*P. putida* G
JDS67PS014	*P. putida*	10	10a	*P. putida* , P-GS2 (*Pseudomonas* sp. NBRC 111134)		*P. putida* G
JDS36PS094	*P. putida*	10	10a	*P. putida* , P-GS2 (*Pseudomonas* sp. NBRC 111134)		*P. putida* G
JDS38PS021	*P. putida*	10	10a	*P. putida* , P-GS2 (*Pseudomonas* sp. NBRC 111134)		*P. putida* G
JDS36PS108	*P. putida*	10	10a	*P. putida* , P-GS2 (*Pseudomonas* sp. NBRC 111134)		*P. putida* G
JDS36PS106	*P. putida*	10	10a	*P. putida*	*P. putida*	*P. putida* G
JDS59PS025	*P. putida*	10	10a	*P. putida* , P-GS2 (*Pseudomonas* sp. NBRC 111134)		*P. putida* G
JDS59PS039	*P. putida*	10	10a	*P. putida* , P-GS2 (*Pseudomonas* sp. NBRC 111134)		*P. putida* G
JDS59PS048	*P. putida*	10	10a	*P. putida* , P-GS2 (*Pseudomonas* sp. NBRC 111134)		*P. putida* G
JDS63PS086	*P. putida*	10	10a	*P. putida* , P-GS2 (*Pseudomonas* sp. NBRC 111134)		*P. putida* G
JDS59PS016	*P. putida*	10	10a	*P. putida* , P-GS2 (*Pseudomonas* sp. NBRC 111134)		*P. putida* G
JDS63PS001	*P. putida*	10	10a	*P. putida* , P-GS2 (*Pseudomonas* sp. NBRC 111134)		*P. putida* G
JDS63PS067	*P. putida*	10	10a	*P. putida* , P-GS2 (*Pseudomonas* sp. NBRC 111134)		*P. putida* G
JDS63PS092	*P. putida*	10	10a	*P. putida* , P-GS2 (*Pseudomonas* sp. NBRC 111134)		*P. putida* G
JDS63PS084	*P. putida*	10	10a	*P. putida* , P-GS2 (*Pseudomonas* sp. NBRC 111134)		*P. putida* G
JDS63PS095	*P. putida*	10	10a	*P. putida* , P-GS2 (*Pseudomonas* sp. NBRC 111134)		*P. putida* G
JDS68PS010	*P. putida*	10	10a	*P. putida* , P-GS2 (*Pseudomonas* sp. NBRC 111134)		*P. putida* G
JDS63PS011	*P. putida*	10	10a	*P. putida* , P-GS2 (*Pseudomonas* sp. NBRC 111134)		*P. putida* G
JDS36PS090	*P. putida*	10	10a	*P. putida* , P-GS2 (*Pseudomonas* sp. NBRC 111134)		*P. putida* G
JDS63PS047	*P. putida*	10	10a	*P. putida* , P-GS2 (*Pseudomonas* sp. NBRC 111134)		*P. putida* G
JDS63PS056	*P. putida*	10	10a	*P. putida* , P-GS2 (*Pseudomonas* sp. NBRC 111134)		*P. putida* G
JDS63PS090	*P. putida*	10	10a	*P. putida* , P-GS2 (*Pseudomonas* sp. NBRC 111134)		*P. putida* G
JDS63PS009	*P. putida*	10	10a	*P. putida* , P-GS2 (*Pseudomonas* sp. NBRC 111134)		*P. putida* G
JDS36PS001	*P. putida*	10	10a	*P. putida* , P-GS2 (*Pseudomonas* sp. NBRC 111134)		*P. putida* G
JDS28PS018	*P. putida*	10	10a	P-GS2 (*Pseudomonas* sp. NBRC 111134)	P-GS2 (*Pseudomonas* sp. NBRC 111134)	*P. putida* G
JDS28PS068	*P. putida*	10	10a	*P. putida* , P-GS2 (*Pseudomonas* sp. NBRC 111134)		*P. putida* G
JDS38PS042	*P. putida*	10	10a	*P. putida* , P-GS2 (*Pseudomonas* sp. NBRC 111134)		*P. putida* G
JDS28PS071	*P. putida*	10	10a	*P. putida* , P-GS2 (*Pseudomonas* sp. NBRC 111134)		*P. putida* G
JDS59PS002	*P. putida*	10	10a	P-GS2 (*Pseudomonas* sp. NBRC 111134)	P-GS2 (*Pseudomonas* sp. NBRC 111134)	*P. putida* G
JDS59PS009	*P. putida*	10	10a	*P. putida* , P-GS2 (*Pseudomonas* sp. NBRC 111134)		*P. putida* G
JDS28PS076	*P. putida*	10	10a	*P. putida* , P-GS2 (*Pseudomonas* sp. NBRC 111134)		*P. putida* G
JDS63PS071	*P. putida*	10	10a	*P. putida* , P-GS2 (*Pseudomonas* sp. NBRC 111134)		*P. putida* G
JDS63PS021	*P. putida*	10	10a	*P. putida* , P-GS2 (*Pseudomonas* sp. NBRC 111134)		*P. putida* G
JDS63PS005	*P. putida*	10	10a	*P. putida* , P-GS2 (*Pseudomonas* sp. NBRC 111134)		*P. putida* G
JDS68PS030	*P. putida*	10	10a	*P. putida* , P-GS2 (*Pseudomonas* sp. NBRC 111134)		*P. putida* G
JDS63PS029	*P. putida*	10	10a	*P. putida* , P-GS2 (*Pseudomonas* sp. NBRC 111134)		*P. putida* G
JDS63PS059	*P. putida*	10	10a	*P. putida* , P-GS2 (*Pseudomonas* sp. NBRC 111134)		*P. putida* G
JDS63PS069	*P. putida*	10	10a	*P. putida* , P-GS2 (*Pseudomonas* sp. NBRC 111134)		*P. putida* G
JDS63PS072	*P. putida*	10	10a	*P. putida* , P-GS2 (*Pseudomonas* sp. NBRC 111134)		*P. putida* G
JDS67PS016	*P. putida*	10	10a	P-GS2 (*Pseudomonas* sp. NBRC 111134)	P-GS2 (*Pseudomonas* sp. NBRC 111134)	*P. putida* G
JDS63PS044	*P. putida*	10	10a	*P. putida* , P-GS2 (*Pseudomonas* sp. NBRC 111134)		*P. putida* G
JDS63PS097	*P. putida*	10	10a	*P. putida* , P-GS2 (*Pseudomonas* sp. NBRC 111134)		*P. putida* G
JDS63PS102	*P. putida*	10	10a	*P. putida* , P-GS2 (*Pseudomonas* sp. NBRC 111134)		*P. putida* G
JDS67PS019	*P. putida*	10	10a	*P. putida* , P-GS2 (*Pseudomonas* sp. NBRC 111134)		*P. putida* G
JDS63PS006	*P. putida*	10	10a	*P. putida* , P-GS2 (*Pseudomonas* sp. NBRC 111134)		*P. putida* G
JDS68PS004	*P. putida*	10	10a	*P. putida* , P-GS2 (*Pseudomonas* sp. NBRC 111134)		*P. putida* G
JDS67PS011	*P. putida*	10	10a	*P. putida* , P-GS2 (*Pseudomonas* sp. NBRC 111134)		*P. putida* G
JDS67PS012	*P. putida*	10	10a	*P. putida* , P-GS2 (*Pseudomonas* sp. NBRC 111134)		*P. putida* G
JDS67PS020	*P. putida*	10	10a	*P. putida* , P-GS2 (*Pseudomonas* sp. NBRC 111134)		*P. putida* G
JDS68PS005	*P. putida*	10	10a	*P. putida* , P-GS2 (*Pseudomonas* sp. NBRC 111134)		*P. putida* G
JDS67PS024	*P. putida*	10	10a	*P. putida* , P-GS2 (*Pseudomonas* sp. NBRC 111134)		*P. putida* G
JDS68PS022	*P. putida*	10	10a	*P. putida* , P-GS2 (*Pseudomonas* sp. NBRC 111134)		*P. putida* G
JDS67PS005	*P. putida*	10	10a	P-GS2 (*Pseudomonas* sp. NBRC 111134)	P-GS2 (*Pseudomonas* sp. NBRC 111134)	*P. putida* G
JDS63PS022	*P. putida*	10	10a	*P. putida* , P-GS2 (*Pseudomonas* sp. NBRC 111134)		*P. putida* G
JDS63PS088	*P. putida*	10	10a	*P. putida* , P-GS2 (*Pseudomonas* sp. NBRC 111134)		*P. putida* G
JDS68PS011	*P. putida*	10	10a	P-GS2 (*Pseudomonas* sp. NBRC 111134)	P-GS2 (*Pseudomonas* sp. NBRC 111134)	*P. putida* G
JDS10PS001	*P. putida*	10	10b	*P. alloputida*	*P. alloputida*	*P. putida* G
JDS59PS013	*P. putida*	10	10b	*P. alloputida*		*P. putida* G
JDS68PS007	*P. putida*	10	10b	*P. alloputida*		*P. putida* G
JDS28PS004	*P. putida*	10	10b	*P. alloputida*		*P. putida* G
JDS28PS009	*P. putida*	10	10b	*P. alloputida*		*P. putida* G
JDS10PS008	*P. putida*	10	10b	*P. alloputida*	*P. alloputida*	*P. putida* G
JDS10PS022	*P. putida*	10	10b	*P. alloputida*		*P. putida* G
JDS36PS024	*P. putida*	10	10b	*P. alloputida*		*P. putida* G
JDS36PS031	*P. putida*	10	10b	*P. alloputida*		*P. putida* G
JDS68PS001	*P. putida*	10	10b	*P. alloputida*		*P. putida* G
JDS10PS011	*P. putida*	10	10b	*P. alloputida*	*P. alloputida*	*P. putida* G
JDS68PS026	*P. putida*	10	10b	*P. alloputida*		*P. putida* G
JDS28PS094	*P. putida*	10	10b	*P. alloputida*	*P. alloputida*	*P. putida* G
JDS10PS025	*P. putida*	10	10b	*P. alloputida*		*P. putida* G
JDS28PS013	*P. putida*	10	10b	*P. alloputida*		*P. putida* G
JDS28PS011	*P. putida*	10	10b	*P. alloputida*		*P. putida* G
JDS38PS001	*P. putida*	10	10b	*P. alloputida*		*P. putida* G
JDS28PS089	*P. putida*	10	10b	*P. alloputida*	*P. alloputida*	*P. putida* G
JDS59PS007	*P. putida*	10	10b	*P. alloputida*		*P. putida* G
JDS28PS003	*P. putida*	10	10b	*P. alloputida*		*P. putida* G
JDS36PS079	*P. putida*	10	10b	*P. alloputida*		*P. putida* G
JDS28PS017	*P. putida*	10	10b	*P. alloputida*		*P. putida* G
JDS38PS034	*P. putida*	10	10b	*P. alloputida*		*P. putida* G
JDS38PS012	*P. putida*	10	10b	*P. alloputida*		*P. putida* G
JDS68PS006	*P. putida*	10	10b	*P. alloputida*		*P. putida* G
JDS28PS033	*P. putida*	10	10b	*P. alloputida*		*P. putida* G
JDS67PS013	*P. putida*	10	10b	*P. alloputida*		*P. putida* G
JDS28PS050	*P. putida*	10	10b	*P. alloputida*		*P. putida* G
JDS59PS008	*P. putida*	10	10b	*P. alloputida*		*P. putida* G
JDS28PS073	*P. putida*	10	10b	*P. alloputida*		*P. putida* G
JDS36PS015	*P. putida*	10	10b	*P. alloputida*		*P. putida* G
JDS36PS033	*P. putida*	10	10b	*P. alloputida*		*P. putida* G
JDS36PS102	*P. putida*	10	10b	*P. alloputida*		*P. putida* G
JDS59PS003	*P. putida*	10	10b	*P. alloputida*		*P. putida* G
JDS59PS004	*P. putida*	10	10b	*P. alloputida*		*P. putida* G
JDS59PS010	*P. putida*	10	10b	*P. alloputida*		*P. putida* G
JDS36PS067	*P. putida*	10	10b	*P. alloputida*	*P. alloputida*	*P. putida* G
JDS36PS095	*P. putida*	10	10b	*P. alloputida*		*P. putida* G
JDS38PS028	*P. putida*	10	10b	*P. alloputida*		*P. putida* G
JDS59PS041	*P. putida*	10	10b	*P. alloputida*		*P. putida* G
JDS36PS051	*P. putida*	10	10b	*P. alloputida*		*P. putida* G
JDS36PS039	*P. putida*	10	10b	*P. alloputida*		*P. putida* G
JDS36PS044	*P. putida*	10	10b	*P. alloputida*		*P. putida* G
JDS36PS007	*P. putida*	10	10b	*P. alloputida*		*P. putida* G
JDS36PS008	*P. putida*	10	10b	*P. alloputida*		*P. putida* G
JDS59PS046	*P. putida*	10	10b	*P. alloputida*		*P. putida* G
JDS59PS040	*P. putida*	10	10b	*P. alloputida*		*P. putida* G
JDS59PS012	*P. putida*	10	10b	*P. alloputida*		*P. putida* G
JDS63PS087	*P. putida*	10	10b	*P. alloputida*		*P. putida* G
JDS59PS038	*P. putida*	10	10b	*P. alloputida*		*P. putida* G
JDS59PS027	*P. putida*	10	10b	*P. alloputida*		*P. putida* G
JDS38PS041	*P. putida*	10	10b	*P. alloputida*		*P. putida* G
JDS63PS065	*P. putida*	10	10b	*P. alloputida*		*P. putida* G
JDS63PS076	*P. putida*	10	10b	*P. alloputida*		*P. putida* G
JDS59PS011	*P. putida*	10	10b	*P. alloputida*		*P. putida* G
JDS38PS040	*P. putida*	10	10b	*P. alloputida*		*P. putida* G
JDS38PS044	*P. putida*	10	10b	*P. alloputida*		*P. putida* G
JDS38PS043	*P. putida*	10	10b	*P. alloputida*		*P. putida* G
JDS59PS042	*P. putida*	10	10b	*P. alloputida*		*P. putida* G
JDS10PS020	*P. putida*	10	10b	*P. alloputida*		*P. putida* G
JDS36PS004	*P. putida*	10	10b	*P. alloputida*		*P. putida* G
JDS28PS051	*P. putida*	10	10b	*P. alloputida*	*P. alloputida*	*P. putida* G
JDS28PS058	*P. putida*	10	10b	*P. alloputida*		*P. putida* G
JDS28PS063	*P. putida*	10	10b	*P. alloputida*		*P. putida* G
JDS28PS100	*P. putida*	10	10b	*P. alloputida*		*P. putida* G
JDS38PS036	*P. putida*	10	10b	*P. alloputida*		*P. putida* G
JDS59PS006	*P. putida*	10	10b	*P. alloputida*		*P. putida* G
JDS28PS092	*P. putida*	10	10b	*P. alloputida*	*P. alloputida*	*P. putida* G
JDS36PS061	*P. putida*	10	10b	*P. alloputida*		*P. putida* G
JDS36PS022	*P. putida*	10	10b	*P. alloputida*		*P. putida* G
JDS63PS039	*P. putida*	10	10b	*P. alloputida*		*P. putida* G
JDS36PS028	*P. putida*	10	10b	*P. alloputida*		*P. putida* G
JDS36PS054	*P. putida*	10	10b	*P. alloputida*		*P. putida* G
JDS36PS068	*P. putida*	10	10b	*P. alloputida*		*P. putida* G
JDS28PS077	*P. putida*	10	10b	*P. alloputida*		*P. putida* G
JDS63PS101	*P. putida*	10	10b	*P. alloputida*		*P. putida* G
JDS38PS005	*P. putida*	10	10b	*P. alloputida*		*P. putida* G
JDS36PS021	*P. putida*	10	10b	*P. alloputida*		*P. putida* G
JDS59PS001	*P. putida*	10	10b	*P. alloputida*		*P. putida* G
JDS63PS010	*P. putida*	10	10b	*P. alloputida*	*P. alloputida*	*P. putida* G
JDS36PS060	*P. putida*	10	10b	*P. alloputida*		*P. putida* G
JDS57PS002	*P. putida*	10	10b	*P. alloputida*		*P. putida* G
JDS63PS007	*P. putida*	10	10b	*P. alloputida*		*P. putida* G
JDS59PS047	*P. putida*	10	10b	*P. alloputida*		*P. putida* G
JDS68PS002	*P. putida*	10	10b	*P. alloputida*		*P. putida* G
JDS36PS084	*P. putida*	10	10b	*P. alloputida*	*P. alloputida*	*P. putida* G
JDS38PS037	*P. putida*	10	10c	*P. monteilii*	*P. monteilii*	*P. putida* G
JDS28PS048	*P. putida*	10	10c	*P. monteilii*		*P. putida* G
JDS28PS088	*P. putida*	10	10c	*P. monteilii*	*P. monteilii*	*P. putida* G
JDS36PS027	*P. putida*	10	10c	*P. monteilii*		*P. putida* G
JDS36PS069	*P. putida*	10	10c	*P. monteilii*	*P. monteilii*	*P. putida* G
JDS28PS099	*P. putida*	10	10c	*P. monteilii*		*P. putida* G
JDS36PS020	*P. putida*	10	10c	*P. monteilii*		*P. putida* G
JDS36PS010	*P. putida*	10	10c	*P. monteilii*		*P. putida* G
JDS36PS019	*P. putida*	10	10c	*P. monteilii*		*P. putida* G
JDS36PS093	*P. putida*	10	10c	*P. monteilii*		*P. putida* G
JDS38PS025	*P. putida*	10	10c	*P. monteilii*		*P. putida* G
JDS59PS031	*P. putida*	10	10c	*P. monteilii*		*P. putida* G
JDS59PS005	*P. putida*	10	10c	*P. monteilii*		*P. putida* G
JDS59PS036	*P. putida*	10	10c	*P. monteilii*		*P. putida* G
JDS38PS003	*P. putida*	10	10c	*P. monteilii*		*P. putida* G
JDS36PS080	*P. putida*	10	10c	*P. monteilii*		*P. putida* G
JDS38PS010	*P. putida*	10	10c	*P. monteilii*		*P. putida* G
JDS36PS023	*P. putida*	10	10c	*P. monteilii*	*P. monteilii*	*P. putida* G
JDS28PS117	*P. putida*	10	10d	*P. asiatica*	*P. asiatica*	*P. putida* G
JDS59PS021	*P. putida*	10	10d	*P. asiatica*, *P. inefficax*		*P. putida* G
JDS36PS018	*P. putida*	10	10d	*P. asiatica*, *P. inefficax*		*P. putida* G
JDS28PS095	*P. putida*	10	10d	*P. asiatica*, *P. inefficax*		*P. putida* G
JDS36PS065	*P. putida*	10	10d	*P. asiatica*, *P. inefficax*		*P. putida* G
JDS36PS006	*P. putida*	10	10d	*P. asiatica*, *P. inefficax*		*P. putida* G
JDS36PS089	*P. putida*	10	10d	*P. asiatica*, *P. inefficax*		*P. putida* G
JDS67PS021	*P. putida*	10	10d	*P. asiatica*, *P. inefficax*		*P. putida* G
JDS38PS008	*P. putida*	10	10d	*P. asiatica*, *P. inefficax*		*P. putida* G
JDS36PS025	*P. putida*	10	10d	*P. asiatica*, *P. inefficax*		*P. putida* G
JDS36PS032	*P. putida*	10	10d	*P. asiatica*	*P. asiatica*	*P. putida* G
JDS10PS012	*P. putida*	10	10d	*P. inefficax*	*P. inefficax*	*P. putida* G
JDS28PS065	*P. putida*	10	10d	*P. asiatica*, *P. inefficax*		*P. putida* G
JDS28PS115	*P. putida*	10	10d	*P. inefficax*	*P. inefficax*	*P. putida* G
JDS28PS067	*P. putida*	10	10d	*P. inefficax*	*P. inefficax*	*P. putida* G
JDS28PS078	*P. putida*	10	10d	*P. asiatica*, *P. inefficax*		*P. putida* G
JDS36PS058	*P. putida*	10	10d	*P. asiatica*, *P. inefficax*		*P. putida* G
JDS36PS071	*P. putida*	10	10d	*P. asiatica*, *P. inefficax*		*P. putida* G
JDS36PS087	*P. putida*	10	10d	*P. asiatica*, *P. inefficax*		*P. putida* G
JDS36PS052	*P. putida*	10	10d	*P. asiatica*, *P. inefficax*		*P. putida* G
JDS36PS050	*P. putida*	10	10d	*P. asiatica*, *P. inefficax*		*P. putida* G
JDS63PS020	*P. putida*	10	10d	*P. asiatica*, *P. inefficax*		*P. putida* G

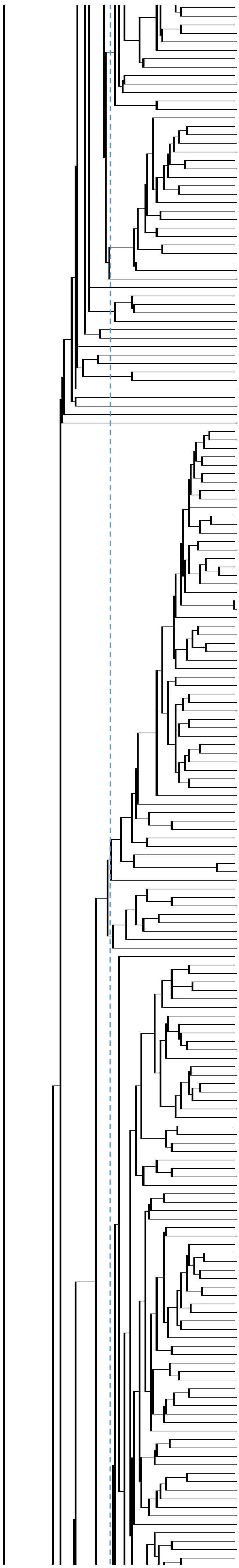

JDS63PS082	*P. putida*	10	10d	*P. asiatica, P. inefficax*		*P. putida* G
JDS63PS083	*P. putida*	10	10d	*P. asiatica, P. inefficax*		*P. putida* G
JDS63PS043	*P. putida*	10	10d	*P. asiatica, P. inefficax*		*P. putida* G
JDS63PS058	*P. putida*	10	10d	*P. asiatica, P. inefficax*		*P. putida* G
JDS68PS034	*P. putida*	10	10d	*P. asiatica, P. inefficax*		*P. putida* G
JDS36PS037	*P. putida*	10	10d	*P. inefficax*	*P. inefficax*	*P. putida* G
JDS10PS016	*P. putida*	10	10d	*P. inefficax*	*P. inefficax*	*P. putida* G
JDS36PS017	*P. putida*	10	10d	*P. inefficax*	*P. inefficax*	*P. putida* G
JDS36PS064	*P. putida*	10	10e	New PS-10	New PS-10	*P. putida* G
JDS36PS066	*P. putida*	10	10f	*P. alloputida*	*P. alloputida*	*P. putida* G
JDS59PS030	*P. putida*	10	10f	*P. alloputida*	*P. alloputida*	*P. putida* G
JDS36PS053	*P. putida*	11	11	New PS-9	New PS-9	*P. putida* G
JDS36PS086	*P. putida*	11	11	New PS-9	New PS-9	*P. putida* G
JDS28PS002	*P. putida*	12	12	*P. fulva*	*P. fulva*	*P. putida* G
JDS38PS009	*P. putida*	12	12	*P. fulva*	*P. fulva*	*P. putida* G
JDS38PS023	*P. putida*	12	12	*P. fulva*		*P. putida* G
JDS49PS001	*P. putida*	12	12	*P. fulva*		*P. putida* G
JDS49PS013	*P. putida*	12	12	*P. fulva*		*P. putida* G
JDS67PS022	*P. putida*	12	12	*P. fulva*		*P. putida* G
JDS68PS012	*P. putida*	12	12	*P. fulva*		*P. putida* G
JDS49PS003	*P. putida*	12	12	*P. fulva*		*P. putida* G
JDS49PS008	*P. putida*	12	12	*P. fulva*		*P. putida* G
JDS49PS012	*P. putida*	12	12	*P. fulva*		*P. putida* G
JDS38PS026	*P. putida*	12	12	*P. fulva*		*P. putida* G
JDS36PS049	*P. putida*	12	12	*P. fulva*		*P. putida* G
JDS38PS011	*P. putida*	12	12	*P. fulva*		*P. putida* G
JDS28PS021	*P. putida*	12	12	*P. fulva*		*P. putida* G
JDS28PS031	*P. putida*	12	12	*P. fulva*		*P. putida* G
JDS36PS098	*P. putida*	12	12	*P. fulva*		*P. putida* G
JDS49PS005	*P. putida*	12	12	*P. fulva*		*P. putida* G
JDS38PS015	*P. putida*	12	12	*P. fulva*	*P. fulva*	*P. putida* G
JDS38PS016	*P. putida*	12	12	*P. fulva*		*P. putida* G
JDS49PS006	*P. putida*	13	13	*P. fulva*		*P. putida* G
JDS59PS043	*P. putida*	14	14	P-GS1 (*P. putida* GB-1)	P-GS1 (*P. putida* GB-1)	*P. putida* G
JDS38PS029	*P. putida*	15	15	*P. juntendi*	*P. juntendi*	*P. putida* G
JDS38PS045	*P. putida*	15	15	*P. juntendi*		*P. putida* G
JDS59PS028	*P. putida*	15	15	*P. juntendi*		*P. putida* G
JDS38PS035	*P. putida*	15	15	*P. juntendi*	*P. juntendi*	*P. putida* G
JDS28PS072	*P. putida*	16	16	New PS-12	New PS-12	*P. putida* G
JDS38PS006	*P. putida*	17	17	*P. fulva*	*P. fulva*	*P. putida* G
JDS59PS022	*P. putida*	18	18	P-GS5 (*P. monteilii* GTC 10897)	P-GS5 (*P. monteilii* GTC 10897)	*P. putida* G
JDS38PS014	*P. putida*	19	19	*P. monteilii*	*P. monteilii*	*P. putida* G
JDS38PS038	*P. putida*	20	20	*P. monteilii*	*P. monteilii*	*P. putida* G
JDS28PS090	*P. putida*	21	21	New PS-18	New PS-18	*P. putida* G
JDS28PS035	*P. putida*	21	21	New PS-18	New PS-18	*P. putida* G
JDS67PS003	*P. putida*	22	22	New PS-15	New PS-15	*P. putida* G
JDS36PS009	*P. putida*	23	23	P-GS8 (*P. putida* PC2)	P-GS8 (*P. putida* PC2)	*P. putida* G
JDS28PS059	*P. putida*	24	24	New PS-11	New PS-11	*P. putida* G
JDS22PS034	*P. putida*	25	25	*P. mosselii*	*P. mosselii*	*P. putida* G
JDS57PS001	*P. putida*	26	26	P-GS2 (*Pseudomonas* sp. NBRC 111134)	P-GS2 (*Pseudomonas* sp. NBRC 111134)	*P. putida* G
JDS02PS006	*P. fluorescens*	27	27a	New PS-6	New PS-6	*P. protegens* SG
JDS02PS010	*P. fluorescens*	27	27a	New PS-6		*P. protegens* SG
JDS22PS011	*P. fluorescens*	27	27a	New PS-6	New PS-6	*P. protegens* SG
JDS10PS033	*P. fluorescens*	27	27a	New PS-6		*P. protegens* SG
JDS22PS012	*P. fluorescens*	27	27a	New PS-6		*P. protegens* SG
JDS10PS031	*P. fluorescens*	27	27a	New PS-6		*P. protegens* SG
JDS22PS007	*P. fluorescens*	27	27a	New PS-6		*P. protegens* SG
JDS02PS024	*P. fluorescens*	27	27a	*P. saponiphila, P. protegens,* New PS-6		*P. protegens* SG
JDS08PS001	*P. fluorescens*	27	27a	New PS-6	New PS-6	*P. protegens* SG
JDS10PS021	*P. fluorescens*	27	27a	New PS-6	New PS-6	*P. protegens* SG
JDS22PS018	*P. fluorescens*	27	27a	New PS-6	New PS-6	*P. protegens* SG
JDS28PS014	*P. fluorescens*	27	27a	*P. saponiphila, P. protegens,* New PS-6		*P. protegens* SG
JDS28PS020	*P. fluorescens*	27	27a	*P. saponiphila, P. protegens,* New PS-6		*P. protegens* SG
JDS02PS016	*P. fluorescens*	27	27a	New PS-6	New PS-6	*P. protegens* SG
JDS28PS028	*P. fluorescens*	27	27a	*P. saponiphila, P. protegens,* New PS-6		*P. protegens* SG
JDS22PS005	*P. fluorescens*	27	27a	*P. saponiphila, P. protegens,* New PS-6		*P. protegens* SG
JDS22PS006	*P. fluorescens*	27	27a	*P. saponiphila, P. protegens,* New PS-6		*P. protegens* SG
JDS22PS010	*P. fluorescens*	27	27a	*P. saponiphila, P. protegens,* New PS-6		*P. protegens* SG
JDS28PS027	*P. fluorescens*	27	27a	*P. saponiphila, P. protegens,* New PS-6		*P. protegens* SG
JDS10PS015	*P. fluorescens*	27	27a	*P. saponiphila, P. protegens,* New PS-6		*P. protegens* SG
JDS10PS017	*P. putida*	27	27a	*P. saponiphila, P. protegens,* New PS-6		*P. protegens* SG
JDS10PS018	*P. fluorescens*	27	27a	*P. saponiphila, P. protegens,* New PS-6		*P. protegens* SG
JDS28PS060	*P. fluorescens*	27	27a	New PS-6	New PS-6	*P. protegens* SG
JDS02PS007	*P. fluorescens*	27	27a	*P. protegens*	*P. protegens*	*P. protegens* SG
JDS08PS003	*P. fluorescens*	27	27a	New PS-6	New PS-6	*P. protegens* SG
JDS02PS009	*P. fluorescens*	27	27a	New PS-6	New PS-6	*P. protegens* SG
JDS02PS020	*P. fluorescens*	27	27a	New PS-6	New PS-6	*P. protegens* SG
JDS08PS004	*P. fluorescens*	27	27a	*P. saponiphila*	*P. saponiphila*	*P. protegens* SG
JDS02PS011	*P. fluorescens*	27	27a	*P. saponiphila, P. protegens,* New PS-6		*P. protegens* SG
JDS28PS024	*P. fluorescens*	27	27a	*P. saponiphila, P. protegens,* New PS-6		*P. protegens* SG
JDS28PS064	*P. fluorescens*	27	27a	*P. saponiphila, P. protegens,* New PS-6		*P. protegens* SG
JDS28PS052	*P. fluorescens*	27	27a	*P. saponiphila, P. protegens,* New PS-6		*P. protegens* SG
JDS67PS009	*P. chlororaphis/fluorescens*	27	27a	New PS-6	New PS-6	*P. protegens* SG
JDS28PS083	*P. fluorescens*	27	27a	New PS-6	New PS-6	*P. protegens* SG
JDS28PS081	*P. fluorescens*	27	27a	New PS-6	New PS-6	*P. protegens* SG
JDS63PS049	*P. fluorescens*	27	27a	*P. saponiphila, P. protegens,* New PS-6		*P. protegens* SG
JDS36PS107	*P. fluorescens*	27	27a	*P. saponiphila, P. protegens,* New PS-6		*P. protegens* SG
JDS28PS075	*P. fluorescens*	27	27a	*P. saponiphila, P. protegens,* New PS-6		*P. protegens* SG
JDS36PS091	*P. fluorescens*	27	27a	*P. saponiphila, P. protegens,* New PS-6		*P. protegens* SG
JDS28PS082	*P. fluorescens*	27	27a	*P. saponiphila*	*P. saponiphila*	*P. protegens* SG
JDS28PS113	*P. fluorescens*	27	27a	New PS-6	New PS-6	*P. protegens* SG
JDS28PS107	*P. fluorescens*	27	27a	*P. saponiphila, P. protegens,* New PS-6		*P. protegens* SG
JDS36PS075	*P. fluorescens*	27	27a	*P. saponiphila, P. protegens,* New PS-6		*P. protegens* SG
JDS10PS014	*P. fluorescens*	27	27a	New PS-6	New PS-6	*P. protegens* SG
JDS36PS016	*P. fluorescens*	27	27a	New PS-6	New PS-6	*P. protegens* SG
JDS36PS096	*P. fluorescens*	27	27b	*P. rhizophila*	*P. rhizophila*	*P. corrugata* SG
JDS67PS006	*P. chlororaphis/fluorescens*	27	27b	New PS-5	New PS-5	*P. corrugata* SG
JDS67PS025	*P. fluorescens*	27	27b	*P. kilonensis*	*P. kilonensis*	*P. corrugata* SG
JDS03PS010	*P. fluorescens*	27	27b	*P. mohnii*	*P. mohnii*	*P. jessenii* SG
JDS68PS023	*P. fluorescens*	27	27b	New PS-2	New PS-2	*P. corrugata* SG
JDS10PS029	*P. fluorescens*	27	27c	*P. lurida*	*P. lurida*	*P. fluorescens* SG
JDS68PS032	*P. fluorescens*	27	27c	*P. lurida*	*P. lurida*	*P. fluorescens* SG
JDS68PS033	*P. fluorescens*	27	27c	*P. lurida*		*P. fluorescens* SG
JDS10PS002	*P. fluorescens*	27	27e	New PS-6	New PS-6	*P. protegens* SG
JDS59PS024	*P. chlororaphis*	28	28a	*P. bubulae*	*P. bubulae*	*P. fragi* SG
JDS59PS020	*P. fluorescens*	28	28a	*P. bubulae*	*P. bubulae*	*P. fragi* SG
JDS59PS029	*P. fluorescens*	28	28a	*P. bubulae*		*P. fragi* SG
JDS59PS034	*P. fluorescens*	28	28a	*P. bubulae*		*P. fragi* SG
JDS67PS002	*P. fluorescens*	28	28a	*P. bubulae*		*P. fragi* SG
JDS59PS035	*P. fluorescens*	28	28a	*P. bubulae*		*P. fragi* SG
JDS59PS033	*P. fluorescens*	28	28a	*P. bubulae*		*P. fragi* SG
JDS59PS019	*P. fluorescens*	28	28a	*P. bubulae*		*P. fragi* SG
JDS02PS012	*P. fluorescens*	28	28b	*P. moraviensis*	*P. moraviensis*	*P. koreensis* SG
JDS02PS013	*P. fluorescens*	28	28b	*P. moraviensis*	*P. moraviensis*	*P. koreensis* SG
JDS02PS015	*P. fluorescens*	28	28b	*P. moraviensis*	*P. moraviensis*	*P. koreensis* SG
JDS02PS018	*P. fluorescens*	28	28b	*P. koreensis, P. moraviensis*, New PS-1		*P. koreensis* SG
JDS36PS038	*P. fluorescens*	28	28b	*P. koreensis, P. moraviensis*, New PS-1		*P. koreensis* SG
JDS28PS066	*P. fluorescens*	28	28b	*P. koreensis, P. moraviensis*, New PS-1		*P. koreensis* SG
JDS10PS004	*P. fluorescens*	28	28b	*P. koreensis*	*P. koreensis*	*P. koreensis* SG
JDS08PS002	*P. fluorescens*	28	28b	*P. koreensis, P. moraviensis*, New PS-1		*P. koreensis* SG
JDS28PS055	*P. fluorescens*	28	28b	*P. koreensis, P. moraviensis*, New PS-1		*P. koreensis* SG
JDS28PS097	*P. fluorescens*	28	28b	*P. koreensis, P. moraviensis*, New PS-1		*P. koreensis* SG
JDS28PS098	*P. fluorescens*	28	28b	*P. moraviensis*	*P. moraviensis*	*P. koreensis* SG
JDS63PS045	*P. fluorescens*	28	28b	*P. koreensis, P. moraviensis*, New PS-1		*P. koreensis* SG
JDS10PS024	*P. fluorescens*	28	28b	*P. koreensis, P. moraviensis*, New PS-1		*P. koreensis* SG
JDS28PS012	*P. fluorescens*	28	28b	*P. koreensis, P. moraviensis*, New PS-1		*P. koreensis* SG
JDS63PS060	*P. fluorescens*	28	28b	*P. koreensis, P. moraviensis*, New PS-1		*P. koreensis* SG
JDS38PS022	*P. chlororaphis/fluorescens*	28	28b	*P. koreensis, P. moraviensis*, New PS-1		*P. koreensis* SG
JDS67PS004	*P. fluorescens*	28	28b	*P. koreensis, P. moraviensis*, New PS-1		*P. koreensis* SG
JDS67PS008	*P. fluorescens*	28	28b	*P. koreensis, P. moraviensis*, New PS-1		*P. koreensis* SG
JDS63PS081	*P. fluorescens*	28	28b	*P. koreensis, P. moraviensis*, New PS-1		*P. koreensis* SG
JDS63PS077	*P. fluorescens*	28	28b	*P. koreensis, P. moraviensis*, New PS-1		*P. koreensis* SG
JDS36PS097	*P. fluorescens*	28	28b	*P. koreensis, P. moraviensis*, New PS-1		*P. koreensis* SG
JDS68PS015	*P. fluorescens*	28	28b	*P. koreensis, P. moraviensis*, New PS-1		*P. koreensis* SG
JDS63PS075	*P. fluorescens*	28	28b	*P. koreensis, P. moraviensis*, New PS-1		*P. koreensis* SG
JDS67PS018	*P. fluorescens*	28	28b	*P. koreensis, P. moraviensis*, New PS-1		*P. koreensis* SG
JDS38PS020	*P. fluorescens*	28	28b	*P. koreensis, P. moraviensis*, New PS-1		*P. koreensis* SG
JDS63PS002	*P. fluorescens*	28	28b	*P. koreensis, P. moraviensis*, New PS-1		*P. koreensis* SG
JDS68PS013	*P. fluorescens*	28	28b	*P. koreensis, P. moraviensis*, New PS-1		*P. koreensis* SG
JDS49PS004	*P. fluorescens*	28	28b	New PS-1	New PS-1	*P. koreensis* SG
JDS08PS005	*P. fluorescens*	28	28b	P-GS13 (*Pseudomonas* sp. NBRC 111117)	P-GS13 (*Pseudomonas* sp. NBRC 111117)	*P. putida* G
JDS28PS023	*P. fluorescens*	28	28b	*P. moraviensis*	*P. moraviensis*	*P. koreensis* SG
JDS28PS085	*P. fluorescens*	28	28b	*P. moraviensis*	*P. moraviensis*	*P. koreensis* SG
JDS28PS105	*P. fluorescens*	28	28b	*P. koreensis, P. moraviensis*, New PS-1		*P. koreensis* SG
JDS28PS008	*P. fluorescens*	28	28b	*P. moraviensis*	*P. moraviensis*	*P. koreensis* SG
JDS67PS001	*P. fluorescens*	28	28b	*P. koreensis, P. moraviensis*, New PS-1		*P. koreensis* SG
JDS28PS057	*P. fluorescens*	28	28b	*P. koreensis, P. moraviensis*, New PS-1		*P. koreensis* SG
JDS28PS084	*P. fluorescens*	28	28b	*P. moraviensis*	*P. moraviensis*	*P. koreensis* SG
JDS63PS032	*P. fluorescens*	28	28b	*P. koreensis, P. moraviensis*, New PS-1		*P. koreensis* SG
JDS63PS031	*P. fluorescens*	28	28b	*P. koreensis, P. moraviensis*, New PS-1		*P. koreensis* SG
JDS63PS089	*P. fluorescens*	28	28b	*P. koreensis, P. moraviensis*, New PS-1		*P. koreensis* SG
JDS28PS056	*P. fluorescens*	28	28b	*P. koreensis, P. moraviensis*, New PS-1		*P. koreensis* SG
JDS63PS078	*P. fluorescens*	28	28b	*P. koreensis, P. moraviensis*, New PS-1		*P. koreensis* SG
JDS63PS073	*P. fluorescens*	28	28b	*P. koreensis, P. moraviensis*, New PS-1		*P. koreensis* SG
JDS63PS098	*P. fluorescens*	28	28b	*P. koreensis, P. moraviensis*, New PS-1		*P. koreensis* SG
JDS36PS057	*P. fluorescens*	28	28b	*P. koreensis, P. moraviensis*, New PS-1		*P. koreensis* SG
JDS63PS046	*P. fluorescens*	28	28b	*P. koreensis, P. moraviensis*, New PS-1		*P. koreensis* SG
JDS63PS034	*P. fluorescens*	28	28b	*P. koreensis, P. moraviensis*, New PS-1		*P. koreensis* SG
JDS63PS015	*P. fluorescens*	28	28b	*P. koreensis, P. moraviensis*, New PS-1		*P. koreensis* SG
JDS63PS018	*P. fluorescens*	28	28b	*P. koreensis, P. moraviensis*, New PS-1		*P. koreensis* SG
JDS36PS092	*P. fluorescens*	28	28b	*P. koreensis, P. moraviensis*, New PS-1		*P. koreensis* SG
JDS36PS101	*P. chlororaphis*	28	28b	*P. moraviensis*	*P. moraviensis*	*P. koreensis* SG
JDS68PS003	*P. fluorescens*	28	28b	*P. koreensis, P. moraviensis*, New PS-1		*P. koreensis* SG
JDS49PS011	*P. chlororaphis/fluorescens*	28	28b	*P. moraviensis*	*P. moraviensis*	*P. koreensis* SG
JDS63PS037	*P. fluorescens*	28	28b	*P. koreensis, P. moraviensis*, New PS-1		*P. koreensis* SG
JDS63PS063	*P. fluorescens*	28	28b	*P. koreensis, P. moraviensis*, New PS-1		*P. koreensis* SG
JDS63PS074	*P. fluorescens*	28	28b	*P. koreensis, P. moraviensis*, New PS-1		*P. koreensis* SG
JDS63PS061	*P. fluorescens*	28	28b	*P. koreensis, P. moraviensis*, New PS-1		*P. koreensis* SG
JDS28PS104	*P. fluorescens*	28	28b	*P. moraviensis*	*P. moraviensis*	*P. koreensis* SG
JDS28PS101	*P. fluorescens*	28	28b	*P. koreensis, P. moraviensis*, New PS-1		*P. koreensis* SG
JDS63PS012	*P. fluorescens*	28	28b	*P. koreensis, P. moraviensis*, New PS-1		*P. koreensis* SG
JDS36PS048	*P. fluorescens*	28	28b	*P. koreensis, P. moraviensis*, New PS-1		*P. koreensis* SG
JDS28PS102	*P. fluorescens*	28	28b	*P. koreensis, P. moraviensis*, New PS-1		*P. koreensis* SG
JDS36PS045	*P. fluorescens*	28	28b	*P. koreensis, P. moraviensis*, New PS-1		*P. koreensis* SG
JDS36PS076	*P. fluorescens*	28	28b	*P. koreensis, P. moraviensis*, New PS-1		*P. koreensis* SG
JDS63PS003	*P. fluorescens*	28	28b	*P. koreensis, P. moraviensis*, New PS-1		*P. koreensis* SG
JDS36PS077	*P. fluorescens*	28	28b	*P. koreensis, P. moraviensis*, New PS-1		*P. koreensis* SG
JDS38PS017	*P. chlororaphis/fluorescens*	28	28b	*P. moraviensis*	*P. moraviensis*	*P. koreensis* SG
JDS63PS079	*P. fluorescens*	28	28b	*P. koreensis, P. moraviensis*, New PS-1		*P. koreensis* SG
JDS36PS074	*P. fluorescens*	28	28b	*P. koreensis, P. moraviensis*, New PS-1		*P. koreensis* SG
JDS38PS013	*P. fluorescens*	28	28b	*P. koreensis, P. moraviensis*, New PS-1		*P. koreensis* SG
JDS63PS048	*P. fluorescens*	28	28b	*P. koreensis, P. moraviensis*, New PS-1		*P. koreensis* SG
JDS67PS007	*P. fluorescens*	28	28b	*P. koreensis, P. moraviensis*, New PS-1		*P. koreensis* SG
JDS67PS017	*P. fluorescens*	28	28b	*P. koreensis, P. moraviensis*, New PS-1		*P. koreensis* SG

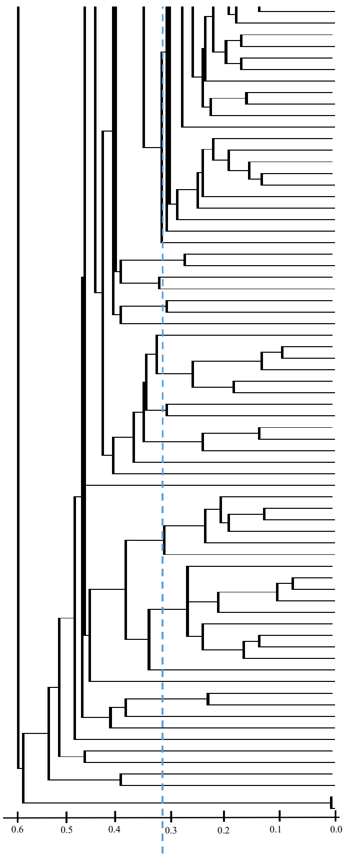

JDS68PS027	*P. fluorescens*	28	28b	*P. koreensis, P. moraviensis*, New PS-1		*P. koreensis* SG
JDS67PS023	*P. fluorescens*	28	28b	*P. koreensis, P. moraviensis*, New PS-1		*P. koreensis* SG
JDS63PS025	*P. fluorescens*	28	28b	*P. koreensis, P. moraviensis*, New PS-1		*P. koreensis* SG
JDS63PS052	*P. fluorescens*	28	28b	*P. koreensis, P. moraviensis*, New PS-1		*P. koreensis* SG
JDS63PS036	*P. fluorescens*	28	28b	*P. koreensis, P. moraviensis*, New PS-1		*P. koreensis* SG
JDS63PS091	*P. fluorescens*	28	28b	*P. koreensis, P. moraviensis*, New PS-1		*P. koreensis* SG
JDS63PS035	*P. fluorescens*	28	28b	*P. koreensis, P. moraviensis*, New PS-1		*P. koreensis* SG
JDS63PS030	*P. fluorescens*	28	28b	*P. koreensis, P. moraviensis*, New PS-1		*P. koreensis* SG
JDS68PS014	*P. fluorescens*	28	28b	*P. koreensis, P. moraviensis*, New PS-1		*P. koreensis* SG
JDS68PS024	*P. fluorescens*	28	28b	*P. koreensis, P. moraviensis*, New PS-1		*P. koreensis* SG
JDS10PS005	*P. fluorescens*	28	28b	*P. koreensis*	*P. koreensis*	*P. koreensis* SG
JDS10PS026	*P. fluorescens*	28	28b	*P. koreensis, P. moraviensis*, New PS-1		*P. koreensis* SG
JDS28PS047	*P. fluorescens*	28	28b	*P. koreensis, P. moraviensis*, New PS-1		*P. koreensis* SG
JDS28PS046	*P. fluorescens*	28	28b	*P. koreensis, P. moraviensis*, New PS-1		*P. koreensis* SG
JDS28PS053	*P. chlororaphis*	28	28b	*P. koreensis*	*P. koreensis*	*P. koreensis* SG
JDS38PS032	*P. chlororaphis*	28	28b	*P. koreensis*	*P. koreensis*	*P. koreensis* SG
JDS10PS027	*P. fluorescens*	28	28b	*P. koreensis, P. moraviensis*, New PS-1		*P. koreensis* SG
JDS59PS014	*P. fluorescens*	28	28b	*P. koreensis, P. moraviensis*, New PS-1		*P. koreensis* SG
JDS63PS096	*P. fluorescens*	28	28b	*P. koreensis, P. moraviensis*, New PS-1		*P. koreensis* SG
JDS63PS019	*P. fluorescens*	28	28b	*P. moraviensis*	*P. moraviensis*	*P. koreensis* SG
JDS36PS104	*P. fluorescens*	28	28b	*P. moraviensis*	*P. moraviensis*	*P. koreensis* SG
JDS59PS037	*P. fluorescens*	29	29	*P. weihenstephanensis*		*P. fragi* SG
JDS59PS026	*P. fluorescens*	29	29	*P. weihenstephanensis*	*P. weihenstephanensis*	*P. fragi* SG
JDS59PS017	*P. fluorescens*	30	30	*P. lundensis*	*P. lundensis*	*P. fragi* SG
JDS59PS045	*P. fluorescens*	31	31	*P. helleri*	*P. helleri*	*P. fragi* SG
JDS49PS007	*P. chlororaphis*	32	32	New PS-3	New PS-3	*P. corrugata* SG
JDS49PS009	*P. chlororaphis*	32	32	New PS-3	New PS-3	*P. corrugata* SG
JDS67PS015	*P. fluorescens*	33	33	New PS-4	New PS-4	*P. corrugata* SG
JDS02PS014	*P. fluorescens*	34	34	New PS-19	New PS-19	*P. putida* G
JDS03PS005	*P. fluorescens*	35	35a	P-GS13 (*Pseudomonas* sp. NBRC 111117)	P-GS13 (*Pseudomonas* sp. NBRC 111117)	*P. putida* G
JDS63PS051	*P. fluorescens*	35	35a	P-GS13 (*Pseudomonas* sp. NBRC 111117)		*P. putida* G
JDS67PS026	*P. fluorescens*	35	35a	P-GS13 (*Pseudomonas* sp. NBRC 111117)	P-GS13 (*Pseudomonas* sp. NBRC 111117)	*P. putida* G
JDS63PS042	*P. chlororaphis*	35	35b	*P. uticensis*	*P. uticensis*	*P. putida* G
JDS63PS070	*P. chlororaphis*	35	35b	*P. uticensis*		*P. putida* G
JDS03PS006	*P. fluorescens*	36	36	New PS-7	New PS-7	*P. syringae* G
JDS28PS106	*P. fluorescens*	36	36	New PS-8	New PS-8	*P. syringae* G
JDS59PS015	*P. viridiflava*	37	37	New PS-22	New PS-22	*P. putida* G
JDS63PS064	*P. fluorescens*	37	37	New PS-22		*P. putida* G
JDS28PS032	*P. viridiflava*	37	37	New PS-22	New PS-22	*P. putida* G
JDS63PS080	*P. oleovorans*	38	38	New PS-21	New PS-21	*P. putida* G
JDS63PS023	*P. viridiflava*	39	39	New PS-20	New PS-20	*P. putida* G
JDS02PS017	*P. putida*	40	40	P-GS11 (*P. monteilii* USDA-ARS-USMARC-56711)	P-GS11 (*P. monteilii* USDA-ARS-USMARC-56711)	*P. putida* G
JDS02PS003	*P. oleovorans*	41	41	New PS-23	New PS-23	*P. oleovorans* G
JDS02PS026	*P. mendocina*	41	41	New PS-23	New PS-23	*P. oleovorans* G
JDS03PS009	*P. mendocina*	41	41	New PS-23	New PS-23	*P. oleovorans* G
JDS28PS118	*P. mendocina*	41	41	P. *toyotomiensis/chengduensis/sihuensis*	P. *toyotomiensis/chengduensis/sihuensis*	*P. oleovorans* G
JDS03PS008	*P. oleovorans*	41	41	*P. oleovorans/indoloxydans*	*P. oleovorans/indoloxydans*	*P. oleovorans* G
JDS02PS005	*P. mendocina*	41	41	*P. guguanensis*	*P. guguanensis*	*P. oleovorans* G
JDS36PS078	*P. oleovorans*	42	42	*P. oleovorans/indoloxydans*		*P. oleovorans* G
JDS68PS018	*P. oleovorans*	42	42	*P. oleovorans/indoloxydans*	*P. oleovorans/indoloxydans*	*P. oleovorans* G
JDS68PS028	*P. oleovorans*	42	42	*P. oleovorans/indoloxydans*	*P. oleovorans/indoloxydans*	*P. oleovorans* G
JDS68PS031	*P. oleovorans*	42	42	*P. oleovorans/indoloxydans*	*P. oleovorans/indoloxydans*	*P. oleovorans* G
JDS68PS025	*P. oleovorans*	42	42	*P. oleovorans/indoloxydans*	*P. oleovorans/indoloxydans*	*P. oleovorans* G
JDS38PS002	*P. oleovorans*	42	42	*P. oleovorans/indoloxydans*	*P. oleovorans/indoloxydans*	*P. oleovorans* G
JDS63PS013	*P. oleovorans*	42	42	P. *toyotomiensis/chengduensis/sihuensis*	P. *toyotomiensis/chengduensis/sihuensis*	*P. oleovorans* G
JDS68PS008	*P. oleovorans*	42	42	P. *toyotomiensis/chengduensis/sihuensis*	P. *toyotomiensis/chengduensis/sihuensis*	*P. oleovorans* G
JDS68PS019	*P. oleovorans*	42	42	P. *toyotomiensis/chengduensis/sihuensis*	P. *toyotomiensis/chengduensis/sihuensis*	*P. oleovorans* G
JDS63PS008	*P. mendocina*	43	43	New PS-24	New PS-24	*P. oleovorans* G
JDS63PS055	*P. alcaligenes*	44	44	*P. resinovorans*	*P. resinovorans*	*P. aeruginosa* G
JDS49PS010	*P. fluorescens*	45	45	*P. carnis*	*P. carnis*	*P. fluorescens* SG
JDS59PS018	*P. fluorescens*	45	45	*P. carnis*		*P. fluorescens* SG
JDS59PS023	*P. veronii*	46	46	*P. veronii*	*P. veronii*	*P. fluorescens* SG
JDS67PS010	*P. fluorescens*	47	47	*P. rhodesiae*	*P. rhodesiae*	*P. fluorescens* SG
JDS22PS013	*P. putida*	48	48	*P. mosselii*	*P. mosselii*	*P. putida* G
JDS28PS114	*P. aeruginosa*	49	49	*P. citronellolis*	*P. citronellolis*	*P. aeruginosa* G
JDS28PS093	*P. aeruginosa*	50	50	New PS-25	New PS-25	*P. aeruginosa* G
JDS10PS010	*P. putida*	51	51	*P. soli*	*P. soli*	*P. putida* G
JDS02PS025	*P. stutzeri*	52	52	*Arthrobacter koreensis*	*Arthrobacter koreensis*[b]	
JDS03PS004	*P. fluorescens*	53	53	*Brevundimonas terrae*		
JDS03PS007	*P. fluorescens*	53	53	*Brevundimonas terrae*	*Brevundimonas terrae*[b]	

[a]Similarity to the closest genomospecies (GS) for *P. putida* G or similarity to the closest genomovar (gv) for *P. stutzeri* G.
[b]Identification for partial sequensce 16S rRNA
