# Supplementary figures and images for: Pseudomonas Species Diversity Along the Danube River Assessed by rpoD Gene Sequence and MALDI-TOF MS Analyses of Cultivated Strains
Source: Front Microbiol. 2020 Sep 2;11:2114. doi: 10.3389/fmicb.2020.02114 (PMC7492575; doi:10.3389/fmicb.2020.02114)

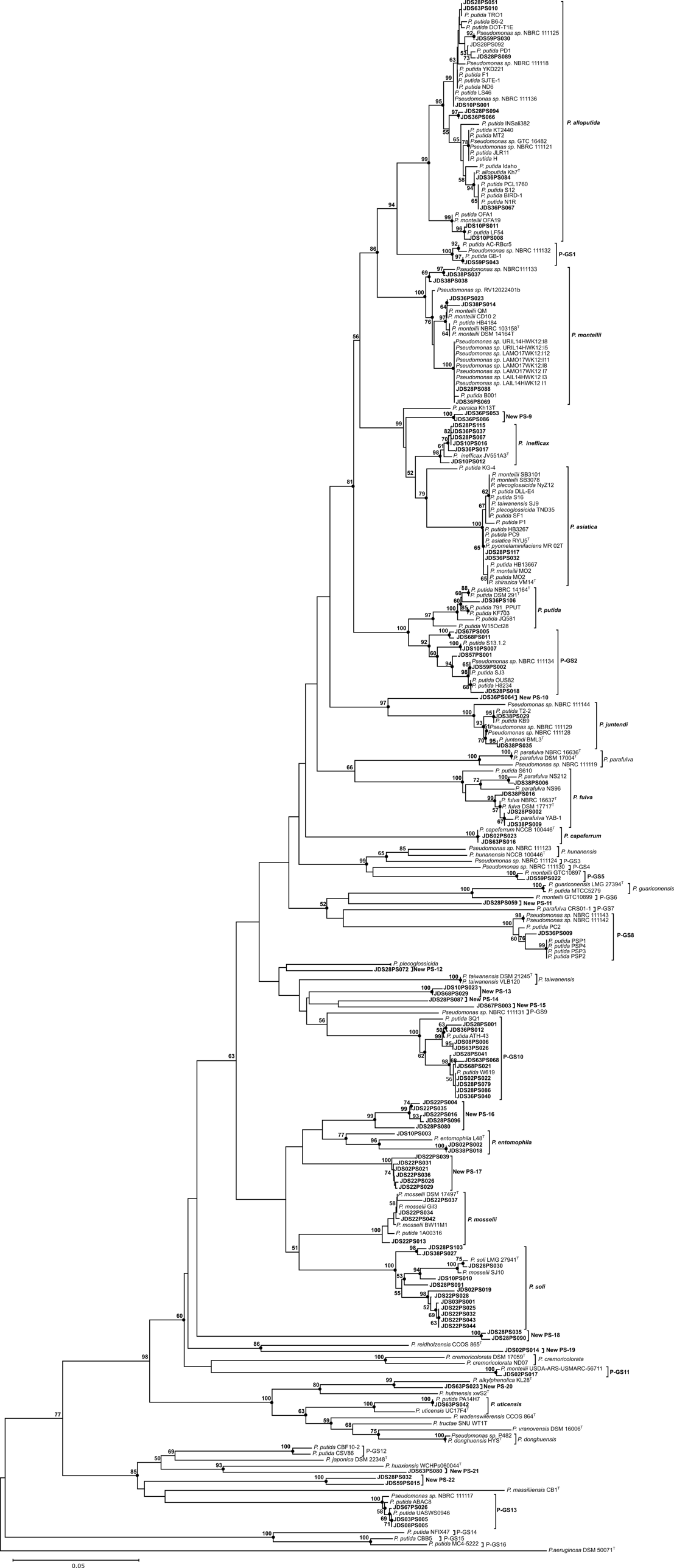

Supplement: FIGURE S3 — Phylogenetic tree of the strains in the P. putida phylogenetic group based on the partial rpoD gene sequence analysis. Distance matrices were calculated by the Jukes-Cantor method. Dendrograms were generated by the neighbor-joining method. P. aeruginosa ATCC 10145T was used as the outgroup. The bar indicates sequence divergence. Percentage bootstrap values of more than 50% (from 1000 replicates) are indicated at the nodes. Filled circles indicate that the corresponding nodes were also obtained in the trees generated with the maximum-likelihood and maximum parsimony methods. [file Image_3.jpeg]

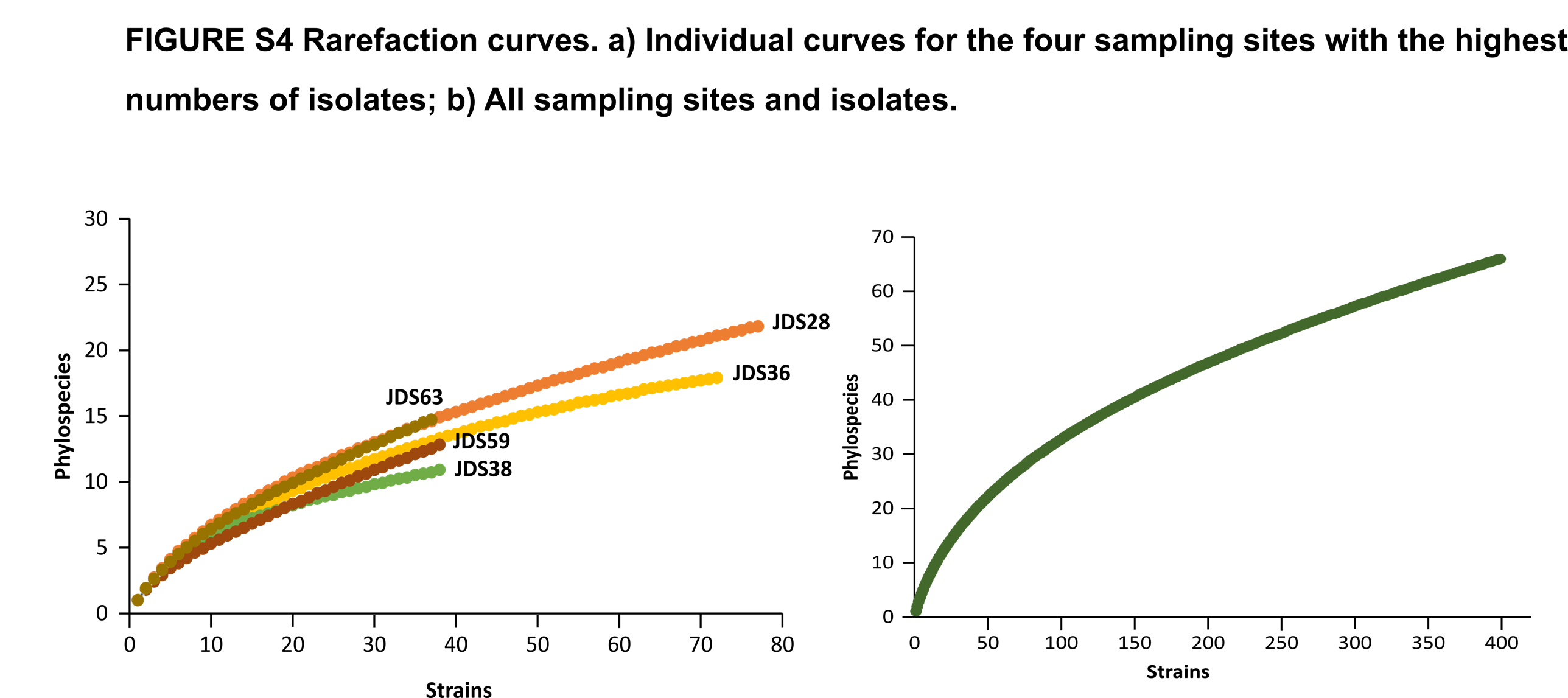

Supplement: FIGURE S4 — Rarefaction curves. (A) Individual curves for the five sampling sites with the highest numbers of isolates; (B) All sampling sites and isolates. [file Image_4.tiff]
