# Supplementary material for: Pseudomonas Species Diversity Along the Danube River Assessed by rpoD Gene Sequence and MALDI-TOF MS Analyses of Cultivated Strains
Source: Front Microbiol. 2020 Sep 2;11:2114. doi: 10.3389/fmicb.2020.02114 (PMC7492575; doi:10.3389/fmicb.2020.02114)
Supplement: TABLE S1 — Location of the 14 sampling points along the Danube River and date of isolation. [file Table_1.DOCX]

**TABLE S1.** **Location of the 14 sampling points along the Danube River and date of isolation.**

| Site | Location | Country | Geographical coordinates* | River Km: Start | Date of Sampling |
| --- | --- | --- | --- | --- | --- |
| JDS02 | Kelheim-gauging station, ds Regensburg | Germany | 48°54'40"N 11°55'47"E | 2415 | 13/08/2013 |
| JDS03 | Geisling power plant | Germany | 48°58'31"N 12°20'46"E | 2354 | 14/08/2013 |
| JDS08 | Oberloiben | Austria | 48°23'03"N 15°31'46"E | 2008 | 18/08/2013 |
| JDS10 | Wildungsmauer, dsVienna | Austria | 48°06'58"N 16°48'01"E | 1895 | 20/08/2013 |
| JDS22 | Budapest downstream – M0 bridge | Hungary | 47°23'21"N 19°00'47"E | 1632 | 26/08/2013 |
| JDS28 | Upstream Drava, us tributary Drava | Croatia/Serbia | 45°35'08"N 18°53'58"E | 1384 | 31/08/2013 |
| JDS36 | Downstream Tisa/Upstream Sava (Belegis) | Serbia | 44°59'23"N 20°17'56"E | 1200 | 04/09/2013 |
| JDS38 | Upstream Pancevo/Downstream Sava | Serbia | 44°50'55"N 20°34'29"E | 1159 | 06/09/2013 |
| JDS49 | Pristol/Novo Selo Harbour | Romania/Bulgary | 44°12'00"N 22°44'17"E | 834 | 13/09/2013 |
| JDS57 | Downstream Ruse/Giurgiu | Bulgaria/Romania | 43°54'43"N 26°03'42"E | 488 | 18/09/2013 |
| JDS59 | Downstream Arges, Oltenita | Romania/Bulgary | 44°03'52"N 26°39'52"E | 429 | 19/09/2013 |
| JDS63 | Siret, Galati | Romania | 45°24'17"N 28°01'54"E | 154 | 22/09/2013 |
| JDS67 | Sulina – Sulina arm | Romania | 45°09'26"N 29°38'46"E | 31 | 25/09/2013 |
| JDS68 | St. Gheorge arm, river delta | Romania | 44°57'08"N 29°29'42"E | 107 | 25/09/2013 |

Geographical coordinates: The samples were taken in a circle of about 500 m around these points.
